# FAIR human neuroscientific data sharing to advance AI driven research and applications: Legal frameworks and missing metadata standards

**DOI:** 10.3389/fgene.2023.1086802

**Published:** 2023-03-13

**Authors:** Aaron Reer, Andreas Wiebe, Xu Wang, Jochem W. Rieger

**Affiliations:** ^1^ Applied Neurocognitive Psychology Lab, Institute for Medicine and Healthcare, Department of Psychology, Oldenburg University, Oldenburg, Germany; ^2^ Chair for Intellectual Property and Information Law, Göttingen University, Göttingen, Germany

**Keywords:** data sharing and re-use, machine learning, open science, FAIR (findable accessible interoperable and reusable) principles, neuroimaging, metadata standards, data protection, privacy law

## Abstract

Modern AI supported research holds many promises for basic and applied science. However, the application of AI methods is often limited because most labs cannot, on their own, acquire large and diverse datasets, which are best for training these methods. Data sharing and open science initiatives promise some relief to the problem, but only if the data are provided in a usable way. The FAIR principles state very general requirements for useful data sharing: they should be findable, accessible, interoperable, and reusable. This article will focus on two challenges to implement the FAIR framework for human neuroscience data. On the one hand, human data can fall under special legal protection. The legal frameworks regulating how and what data can be openly shared differ greatly across countries which can complicate data sharing or even discourage researchers from doing so. Moreover, openly accessible data require standardization of data and metadata organization and annotation in order to become interpretable and useful. This article briefly introduces open neuroscience initiatives that support the implementation of the FAIR principles. It then reviews legal frameworks, their consequences for accessibility of human neuroscientific data and some ethical implications. We hope this comparison of legal jurisdictions helps to elucidate that some alleged obstacles for data sharing only require an adaptation of procedures but help to protect the privacy of our most generous donors to research … our study participants. Finally, it elaborates on the problem of missing standards for metadata annotation and introduces initiatives that aim at developing tools to make neuroscientific data acquisition and analysis pipelines FAIR by design. While the paper focuses on making human neuroscience data useful for data-intensive AI the general considerations hold for other fields where large amounts of openly available human data would be helpful.

## 1 Introduction

Making data publicly available is considered beneficial for scientific research in many respects including improving reliability of results by increasing transparency and quality, increasing efficiency of the (public) money spent, accelerating innovation by enhancing interdisciplinarity, and enabling the use and development of new analysis techniques (Milham et al., 2018; Niso et al., 2022). For these reasons, opening up the currently mostly closed scientific research, e.g., by encouraging data sharing (among other research products and practices) is one of today’s pressing issues.

Replication and reproducibility issues recently elicited growing concerns in the biomedical and life sciences over the credibility of claims raised in scientific studies and the economic efficiency of research. The OPEN SCIENCE COLLABORATION (2015) tried to replicate 100 highly influential studies published in top-tier psychology journals and found that in only 36% of these studies statistical significance of the results could be reproduced. Glasziou and Chalmers (2018) argue that due to fundamental deficiencies in the design and conduct of studies in clinical research, globally around 85% of the money being spent is wasted because many findings cannot be reproduced, nor can the respective data be re-used. Moreover, the authors concluded that many findings can or should not be implemented into practice due to their low reliability. Similarly, a meta-analysis of past studies on the cost of non-reproducible research has revealed that in the US over 50% of the preclinical research cannot be reproduced and therefore complicates cumulative knowledge acquisition (Freedman et al., 2015). According to the authors, this amounts to approximately 28 billion US dollars per year being misspent in the US alone. Today a common notion is that, among others, open sharing of data and research products is one important measure to make research more efficient in its resource use (Niso et al., 2022). The 2020 EU scoping report on “reproducibility of scientific results in the EU” (Europäische Kommission et al., 2020) and the 2019 report of the US National Academies of Sciences, Engineering, and Medicine on “Reproducibility and replicability in science” (National Academies of Sciences, 2019) list, among others, data sharing as one important scientific practice to enhance reproducibility and replicability. This includes, training for data sharing, the establishment and improvement of data sharing plans in publicly funded research, support for data sharing, the resolution of data sharing problems, and FAIRification of shared data. Sharing is also considered a measure to trigger a change in scientific practice from closed research to open sharing of research products to increase the quality and transparency of research practices.

One way to estimate an increase in efficiency of resource use by data sharing is to estimate potential monetary savings. This is of relevance as most research in public institutions is financed by public money. Employing a bibliometric analysis of the re-use of five openly shared large scale neuroimaging datasets provided by the International Neuroimaging Data-sharing Initiative (INDI, Mennes et al., 2013) Milham and others estimate savings of 900 million up to 1.7 billion US dollars compared to re-acquisition of the data for each of the approximately 900 papers published on the basis of these datasets (Milham et al., 2018). Likewise, the European Commission has issued a report in 2019 suggesting that better research data management would save 10.2 billion euros per year in Europe (European Commission and Directorate-General for Research and Innovation, 2019). They even argue that potentially the gain would be even bigger (up to an additional 16 billion euros) due to the generated innovation, e.g., faster accumulation of knowledge and potential savings of money spent on data acquisition.

Beyond improving credibility, reliability, and efficiency of research, individual researchers may personally benefit as well from sharing their data. Data sharing can increase their visibility and reputation by licensing the data and making it a citable object. This offers new opportunities for publications and can increase the number of citations, raise media attention, open new collaborations with researchers who do not belong to the narrow group of the individual research field, and finally can offer new funding and position opportunities (Markowetz, 2015; McKiernan et al., 2016; Allen and Mehler, 2019; Hunt, 2019; Niso et al., 2022; Nosek et al., 2022). However, it is important to note, that practices such as data sharing or proper description of the data through metadata imposes additional work for the individual researcher. For this reason, it is important to facilitate the implementation of these practices into workflows in order to lift some weight off the shoulders of the individual researcher. In other words, usability must be a critical aspect of tools for sharing or organizing data.

In the light of the issues with closed research and the potential advantages of sharing data and other research products the general reluctance of researchers to share their data appears surprising (Houtkoop et al., 2018). Recently, however, the importance of data sharing and research data management (RDM) moved from a small community of open science enthusiasts into the focus of funding agencies and journals as policy reinforcers to address these issues. Funding agencies are beginning to implement a top-down strategy for publicly funded research to expand data sharing for more efficient data use and accessibility of research results (de Jonge et al., 2021; Niso et al., 2022). Some funding agencies require RDM plans, openly accessible publications, and dissemination plans beyond journal publications. In addition, an increasing number of journals offer open access options and require authors to make their data publicly available (Niso et al., 2022). In parallel stakeholder institutions like the Organization for Human Brain Mapping (OHBM)^1^, the International Neuroinformatics Coordinating Facility (INCF)^2^ and the Chinese Open Science Network (COSN)^3^, coordinate the development of data standards and best practices for open and FAIR research data management.

Data sharing in standardized data formats and enriched with metadata are important requirements for novel data-driven Artificial Intelligence (AI) analysis techniques. AI technologies are expected to propel and transform scientific research in the near future and are meanwhile key technologies in medical research, diagnostic procedures, etc. They learn generalizable structure in complex data which is unrecognizable to humans and make it possible to predict e.g., disease risks or cognitive functions in new data. This development is supported by the increasing capacity of computing machines that enable more complex computations on ever-growing data sets. However, many AI algorithms estimate extremely complex models from the data. This requires huge amounts of data. The limited amount of available data within single labs (with known data structure and metadata) and the limited amount of well structured, meta-data annotated, and exhaustively documented publicly available data is a common bottleneck for the reliable application of complex but powerful AI methods. Therefore, data sharing and making experimental data interoperable (i.e., common data and annotation standards help to make the data computer interoperable) has become an important goal for the neuroimaging community.

Several fields in life science and medicine have recognized the potential of publicly available data early and started large scale initiatives to make data collected in individual labs accessible for other research groups in order to maximize the scientific benefits. The forerunners were the Human Genome Project^4^, launched in 1990, in which the Bermuda principles were developed. These required the timely sharing of annotated sequence data (Collins et al., 2003). This policy initially boosted progress in genomic research and in related fields such as computer science and AI based data analysis (Rood and Regev, 2021). Hence it fostered interdisciplinary research approaches, digitization of life science research and the development of novel analysis methods (Gibbs, 2020). Over the years, the increase in size and complexity of available data, the lack of data standards, the scattering of data across various databases, and data privacy issues, in particular when the genetic data were enriched with “phenotype” metadata, have triggered a re-thinking of the current relatively unstructured sharing approach. This re-thinking was mainly due to the fact that it became more and more evident that this unstructured approach likely has a negative impact on the usability and usefulness of the shared data in current and future usage scenarios (Powell, 2021). Moreover, while the domain of genetics developed a relatively generous and open data culture, recent developments indicate a return to closed data policies with reluctance to share data or only under certain conditions, for example, data sharing policies in the commercial sector and in China (Koch and Todd, 2018; Chen and Song, 2018; PIPL Art. 38–43&53). This closed policy cuts international public genetics research off from huge data sources. In neuroscience, the later funded Human Connectome Project (launched in 2009) and the EU Human Brain Project (launched in 2013) also collect massive amounts of complex datasets consisting of diverse data types (e.g., brain imaging data recorded with different measuring techniques or devices, behavioral data, data about the experimental paradigm, genetic data, bio samples, clinical diagnostics, psychological testing, etc.). This was done to provide datasets, that enable tackling a range of research questions by different researchers, even questions unrelated to the original study. In general, acquiring more diverse data in an experiment, exceeding those needed for the original research question, would help to increase the efficiency of data re-use. While some efforts have been made to create publicly open databases to make the data accessible, common standards on how to store such datasets are only emerging (e.g., Teeters et al., 2015; Gorgolewski et al., 2016).

Publicly open databases which contain well described and standardized datasets help to make the data better understandable not only for humans but also for computers. Accordingly, such datasets can serve as training data for the development of new analysis approaches but also as realistic benchmark datasets to compare the performance of novel AI algorithms. Well-structured data enhanced with metadata and many accessory observational data are also attractive for researchers who have no access to the expensive experimental infrastructure, be they from different fields, like computational neuroscientists, developers of AI algorithms or experts from countries or research sites with less financial resources. In these cases, data sharing can make science more interdisciplinary and diverse by adding hitherto excluded modelers, methods developers, and researchers without access to neuroimaging resources to a research community.

In sum, data sharing offers benefits for the individual researcher as well as research communities besides improving transdisciplinary integration of research and thereby enhancing its development. So why is so little of the myriads of data produced in biomedical and life science publicly shared (Houtkoop et al., 2018)? There are many possible reasons, ranging from motivation and literacy to infrastructural problems at the level of FAIRification as well as legal and ethical issues, that create uncertainty under which conditions human research data can be shared and with whom (Paret et al., 2022). In this paper we will focus on two related issues. First, we want to outline the heterogeneous legal frameworks with respect to data privacy in different geopolitical zones. The focus of this analysis will be on comparing the goals of the frameworks and to explicate the constraints they impose on sharing of sensitive human data. Second, we discuss approaches for data organization and metadata annotation in the domain of neuroscience. In other words, standardized vocabularies or ontologies for turning data into meaningful and interpretable information. Finally, we will highlight initiatives and tools, that were developed to help the individual researcher to practically implement data sharing into their workflows.

## 2 Challenges for useful data sharing

Although data sharing is generally regarded as a good and desirable practice, it creates technical as well as ethical and legal challenges. Depending on how well these are met, the effects of data sharing can range from useful to harmful. As always, a good intent does not guarantee a good deed. Two big challenges to the useful sharing of human neuroimaging data will be highlighted in the following.

A first challenge for sharing data from humans arises when they include personal data or become personalizable (e.g., when biometric data such as genetic information or pictures of a person are included). Then legal and ethical restrictions may require higher control levels for data sharing. Complications arise from the fact that legal and ethical data protection levels vastly differ between states and cultures around the world and that it is often unclear what combination of features can make the data personalizable. A second challenge arises from the fact that data from experiments with human participants are oftentimes complex, leaving the experimenter a lot of freedom with respect to organizing and describing them. Then metadata, data describing data, is required to make the data useful and interpretable for other researchers or automated analysis pipelines. We call this the metadata description challenge.

It should be noted that technical challenges like provision, maintenance and setting up of databases, and the technical implementation of safeguards for these repositories etc. is not in the scope of the paper. Moreover, in this article we focus on neuroimaging data. However, some of the points discussed, in particular the legal frameworks, also apply to other types of human data, like genetic data.

### 2.1 Legal and ethical frameworks around data sharing

Privacy issues can arise when the human neuroimaging data allow for re-identification of the person from whom they were recorded. By re-identification we mean, that the data may provide information, that makes it possible to tell whether it was recorded from let’s say Jane Roe or Henry Wade. For example, anatomical magnetic-resonance-imaging (MRI) scans can contain an image of the face which might allow for re-identification of the person. Schwarz and others demonstrated that individual subjects could be re-identified by matching the faces reconstructed from MR-scans with pictures from the subjects that originated from social media (Schwarz et al., 2019). Another study showed that blurring the face in the MRI-scan may not be sufficient to prevent re-identification. Using Generative Adversarial Networks, it was shown that blurred faces could be reasonably well reconstructed to allow for re-identification. However, completely removing the facial features from the anatomical MRI scans greatly reduced the success of the method (Abramian and Eklund, 2019). In the field of neuroimaging this debate is most relevant for high resolution structural imaging techniques that can provide anatomical images, such as certain magnetic resonance imaging techniques. Electrophysiological recordings, such as EEG and MEG, or fNIRS do not provide detailed anatomical information. For that reason, data from these devices are less likely to be re-identifiable (Jwa and Poldrack, 2022; White et al., 2022). There is an ongoing discussion, however, to what extent neuroimaging data in general contain individual signatures, similar to genetic data. It has thus been suggested to consider them as a kind of biometric data, i.e., some data that is not alone identifiable but sufficient to single out data from an unidentified individual X in a group of datasets (Bannier et al., 2021). Whether the fear that human neurophysiological data allow for direct re-identification or singling out and subsequent identification of an individual is in general realistic or whether these are overly conservative assumptions still needs to be shown (Jwa and Poldrack, 2022). Moreover, it is not clear how future developments like increasing data availability, complexity, and progress in AI techniques contribute to the problem.

Such considerations are necessary because privacy and data protection laws across jurisdictions offer protection against processing of information from which a person may be re-identified. Therefore, a basic prerequisite of shared neuroimaging data and accompanying metadata is that the natural person from whom it was recorded cannot be re-identified. This can create a tension between the desire to have rich datasets with lots of metadata describing the individual (including phenotypic data), and privacy protection. Potential privacy breaches can have different consequences in different legal, ethical and cultural regions because data privacy and data protection is weighted very differently across regions and respective jurisdictions. This can make sharing of neuroimaging but also other “biometric” or “identifiable” data across borders very difficult (Eke et al., 2022). The main existing legal frameworks appear to revolve around three agents: a natural person who donates data, private institutions with commercial interests in the donated data, and governmental institutions with various goals regarding the data. Below, we provide an overview of the laws/regulations from three representative jurisdictions EU, United States, and China. These are the regions with the largest data resources and they span a spectrum of regulatory frameworks in which the different and potentially conflicting interests of the three agents are balanced and weighted differently. Readers looking for a quick overview of the regulations relevant for sharing human research data internationally can refer to Table 1. Details and sources to each point are provided in the text. A list of points researchers should consider when planning to acquire data for sharing is provided in Table 2 at the end of the chapter.

**TABLE 1:** Overview of data protection regulations for publicly funded research.

Aspect	EU	United States	China
Relevant laws and regulations	GDPR and local data protection laws as instances of it.	Dependent on applicable regulator: e.g., HIPAA, Common Rule, special rules.	CSL, DSL, PIPL, CCC, and field specific regulations by MOST
What is protected	All processing of identifiable, pseudonymized, or special personal data. Anonymized data are exempted.	Common Rule: only identifiable private information collected during research.	Personal information (includes sensitive information, such as biometric and medical health data) and data sovereignty of China.
		HIPAA: personal health related data.	
What is personal information	Any information related to identified or identifiable natural person	Common Rule: Information from which the identity of the subject be readily ascertained	Information related to identified or identifiable natural persons.
		HIPAA: individually identifiable health information.	
Measures of responsible person (e.g., researchers) to protect personal data	Pseudonymization (e.g., replace identifiable information with code), anonymization (no identifiable and sensitive information)	Common Rule: Unclear	De-identification, anonymization (impossible to identify person)
		HIPAA: de-identification e.g., by Safe Harbor Method (similar to pseudonymization) and DUA	
Consent required for	All processing (here sharing) of personal, pseudonymized and sensitive data. Extended consent possible. For non-sensitive data other legal grounds Art. 6 I	Common Rule: Broad consent. Secondary use without consent.	Separate consent for different processing purposes. Sensitive data additionally require special purpose. New purposes require new consent.
		HIPAA: written informed consent for data sharing or DUA	
IRB required	Yes and legal assessment	Yes	IRB not mentioned. Sharing might be restricted by state institutions (e.g., genetic data).
With whom can adequately protected data be shared	Researchers in EU and adequacy region. Consent and DUA may allow widening scope.	Common Rule: policy evolving. HIPAA rules sufficient.	Sharing outside mainland requires several safety measures and local safeguard.
		HIPAA: With consent and/or DUA no restriction.	

We would like to point out that the following section only provides an informative overview with pointers to regulations we considered relevant for the comparison. They should not be considered as legal advice.

#### 2.1.1 The European Union

In the EU the General Data Protection Regulation (GDPR) came into effect in 2018. The GDPR was a major step to harmonize the legal regulations for acquisition, processing, and sharing of personal data across the jurisdictions of the Member States. This was necessary to ensure free movement of data between EU member states and states providing comparable data protection. It is based on *codified legal principles* relating to the protection of personality and most parts were implemented in the laws of the EU member states before. It is directly applicable as a regulation in all Member States without the need for further national implementation. However, there are supplementary national and local regulations specifying the rules and the GDPR is open for deviating national legislation in some cases. In a sense the GDPR follows the European tradition of the enlightenment as it aims to put the individual at the center and it follows the tradition of civil law. One motivation contributing to the design of the GDPR was to empower the individual against economic interests of companies which often consider the acquired data as their property which they can use without further accountability. The examples for questionable or unethical acquisition, use and (not-) sharing of user data by the big tech companies are legion (e.g., The European Data Protection Supervisor (EDPS)^5^, 2020; Koch and Todd, 2018; Kurtz et al., 2022; Spector-Bagdady, 2021). In addition, the opaque handling of collected data, research practices and goals, created suspicions that these practices raise barriers for research and that egoistic economic goals of research can severely conflict with the interests of the individual as well as society. We will briefly review the regulations relevant for scientific data sharing in the following sections.

The GDPR’s enormous impact is due to the broad scope that reaches beyond institutions established in the EU. It applies to any processing (e.g., analysis and sharing) of personal data in the context of the activities of a *data controller* (person who has control over the data) or a *data processor* (person who processes the data), regardless of whether these activities take place in the Union or not (GDPR Art. 3 (1)). The GDPR also restricts collection and processing of personal data by states. In short, the GDPR provides regulations for the protection of personal data of natural persons by establishing binding principles (e.g., transparency, purpose limitation and data minimization, GDPR Art. 5) and by defining a set of lawful processing purposes (GDPR Art. 6). One way to implement legal processing is to obtain consent from the person who donates data (data subject). The GDPR also defines rights of data subjects (GDPR Art. 12–23), and mechanisms to enforce their rights (GDPR Art. 77–84).

The GDPR defines *personal data* broadly as “any information relating to an identified or identifiable natural person”, the data subject (GDPR Art. 4 (5)). One measure to protect personal data is to *pseudonymize* it (GDPR Art. 25), meaning that the data are processed in a way that they cannot be directly related to the data subject. This can be achieved, for example, by separating all personal information, that would allow re-identification, e.g., data to handle the compensation for participation like name, address, bank account etc., from the data to be processed. The link between data and personal information is stored in a coding list which is kept separate from the data. Pseudonymization is a safeguard for sharing that is provided in other regulations too (see Sections 2.1.2, 2.1.3) and in practice most neuroimaging labs already implement such a policy. Moreover, the coding list would be in the hands of the data controller, who determines the means and purposes of the data processing, but it would not be accessible to the data processor. This is not always possible, e.g., when the data controller and the data processor are the same person. However, there are ways to deal with such problems, e.g., by handing coding list and personal information to another trustworthy person. Importantly, the coding list must not be shared and a third-party data processor must not gain access to the content of the coding list. It should be noted that pseudonymized data are still in the scope of the GDPR as they can be associated with a data subject by means of other information (e.g., the coding list, Recital 26). Conversely, *anonymous* data, which cannot be related to a natural person, is not covered by the GDPR (Recital 26), meaning that processing of anonymized data is outside the scope of the GDPR. However, this is not true for the processing up to the point of anonymization, for which a legal ground is still necessary. The GDPR does not suppose that means for personal data protection are perfect and unbreakable. Therefore, it adopts a risk-based protection assessment. The risk of re-identification or other misuse of the data should be minimized by considering state-of-the-art technology, but the data controller should also consider the costs for protection and re-identification, as well as the likelihood and severity of risks arising for the natural person from re-identification (GDPR Art. 25, 32, Recital 26).

As the GDPR promotes privacy by design and default (GDPR Art. 25) it has been argued that personal data cannot be shared with other researchers under the GDPR and that the GDPR therefore poses an obstacle for free international dataflow and hence scientific research (Eke et al., 2022). Unfortunately, this is a widely adopted misconception. The GDPR weights the value of scientific research and offers a range of derogations from the strict protection of personal data for *scientific research and academic expression* (GDPR Art. 85, Art. 5 (1) (b), (e)). However, some safeguards (GDPR Art. 89) must be met. The European Data Protection Supervisor (EDPS 2020)^6^ lists transparency and being in the public interest as central features of scientific research. Moreover, the safeguards that need to be implemented include explicit informed consent to the sharing of personal data and independent ethical oversight, e.g., by an ethics committee. Personal data can be processed to make them suitable for archiving in public interest, meaning they can be pseudonymized and made available in research data repositories in pseudonymized form. Moreover, the data can be processed for scientific, historical, and statistical purposes (GDPR Art. 89) and for other purposes than those for which they were initially collected if consent was collected and recognized ethical standards for scientific research are met (GDPR Recital 33, 50). Privacy by design and default can be supported by Codes of Conduct like the “Code of Conduct on privacy for mobile health applications”^7^ though that has not yet been adopted. Moreover, the position paper “A preliminary opinion on data protection and scientific research.” by the EDPS (2020)^8^ provides some advice for the interpretation of the GDPR in that respect.

The GDPR, like other regulations (see Sections 2.1.2, 2.1.3), puts particularly strong restrictions on the processing of *special categories of data* (e.g., health data or biometric data, Art. 9 GDPR). Some processing purposes are allowed and explicit consent for the processing is required (Art. 9 GDPR). But the GDPR also acknowledges the importance of science and research for society and provides some privileges for research purposes, to balance research with the rights of the individual (see Wiebe, 2020) and permits derogations to the prohibition of the processing of special data in accordance with GDPR Art. 89. Of special relevance are processing permissions for scientific purposes in Art. 9 (2) (j) GDPR that are specified by national legislation. For example, in Germany, the weighing of interests is a prerequisite for lawful processing (§ 27 German Data Protection Statute, BDSG). Article 7 (2) (h) of GDPR defines permissions for medical and (public) health related processing. However, specific measures to safeguard the fundamental rights and interests of the data subject must be implemented. For neuroimaging data sharing, explicit consent, mechanisms for access control and contracts in the form of data use agreements have been suggested (Bannier et al., 2021; Staunton et al., 2022). The exact scope of these permission with respect to the development and use of AI systems in the health sector has still to be developed, in connection with appropriate safeguards.

In the context of scientific research, data fulfilling the outlined requirements of the GDPR can and should be freely exchanged between researchers in the EU member states and those states with an adequacy decision, which means that they are recognized to offer data protection at a comparable level as the GDPR (see here^9^ for a list of countries for which such adequacy decisions have been made). The Data Governance Act2022^10^ seeks to enhance *data sharing* by removing technical and organizational obstacles to data sharing and provide a secure infrastructure for data sharing within the EU. It includes the promotion of the development of data intermediation services and the development of arrangements to facilitate data use on altruistic grounds, i.e., to make data available voluntarily, without reward, to be used in the public interest. E.g., Art. 25 of the Data Governance Act foresees the development of a European data altruism consent form, which shall allow the collection of consent or permission across Member States in a uniform format. Moreover, the European Commission issued plans to build a European Health Data Space which provides individual persons control over their health data in concordance with the GDPR (Directorate-General for Health and Food Safety, 2022)^11^. However, currently, due to a very restrictive decision of the European Court of Justice^12^, transfer of personal data to third countries are very difficult to pursue lawfully with very high requirements on safeguards in the target country and their practical effectiveness. This applies to each country for which no adequacy decision, as stated above, exists, including countries like China and the United States. On the political level, efforts are underway to establish a renewed “safe harbor” for transfers to the U.S. However, this does not mean that personal or special category data cannot be shared with researchers in countries outside the EU or if no adequacy decision exists. For the transfer of special data to a third country without adequacy decision, explicit consent to the transfer by the data subject can be a potential legal basis if the transfer is not done on a regular basis (GDPR Art 49; EDPS, 2020^13^).

In sum, the GDPR defines a legal framework for the processing and transfer of personal data that aims to protect the individual and harmonize the legal frameworks across member states in order to simplify privacy protection and data exchange between states. It establishes as world-wide “gold standard” and serves as a blue print for most recently developed personal data protection laws (Greenleaf, 2022), among many others in multiple US-states (California, Wyoming, Ohio New York), in Canada, Brazil, and in some parts for the recently enacted Personal Information Protection Law of China.

#### 2.1.2 The United States of America

In comparison to other nations the US has relatively weak personal data protection laws and data transfer legislations (Pernot-Leplay, 2020; Jwa and Poldrack, 2022). However, at the same time, in the United States the situation is complex and follows the tradition of *case law* that aims to regulate actions of agents. The regulations under which data are shared have been developed by several bodies with different fields of competence. Consequently, the regulation under which human data is shared might depend on the goal of the research (e.g., FDA for medical device development) and where it was collected (e.g., HIPAA for healthcare providers or the Common rule which defines a baseline standard for almost any government-funded research in the US). In addition, specific rules of funding bodies may apply. These regulations were developed to support the basic ethical principles of respect for persons (autonomy supported by informed consent), beneficence (assessment of risks and benefits), justice (selection of participants) stated in the Belmont Report (National Commission for the Protection of Human Subjects of Biomedical and Behavioral Research, 1979)^14^. The regulations are not laws as they were developed by federal regulatory bodies but not by congress (Kulynych, 2007; Clayton et al., 2019). Therefore, sanctioning of violations of the regulations is done by the regulatory departments of the funding bodies. Individual research participants may have only limited means for legal action (Spector-Bagdady, 2021). Generally, this may be sufficient for publicly funded neuroimaging research but it has been questioned if the Common Rule is sufficient to guarantee privacy rights to research subjects in the private sector, for example, for companies who collect genetic data to build database for commercial secondary use (Koch and Todd, 2018; Meyer, 2020). The existing situation leaves a large space for a field of unregulated research on human subjects and data processing/brokering, e.g., in privately funded research (Price and Cohen, 2019; Price et al., 2019; Meyer, 2020). The situation is sufficiently complex that we can provide here only a coarse overview. More in depth reviews are provided, for example, by Kulynych (2007) and Spector-Bagdady (2021). In the following, we will briefly go through a few aspects of the above-mentioned regulations relevant for data sharing.

The most basic fallback regulation if no other specific regulation applies (see below) is the *Common Rule* (45 CFR 46), it was defined by the Department of Health and Human Services and has been adopted by a number of federal agencies that fund or conduct research. In addition, institutions not covered may voluntarily submit an assurance to comply with it. Virtually all academic research institutions in the US are covered by the Common Rule under these premises. However, there is research on humans that is not covered by the Common Rule because it is exempt, the institutions are not federally funded, do not want to provide an assurance, or because they are covered by a different regulation (Meyer, 2020). The Common Rule has a broad *action-oriented definition of research* as “a systematic investigation, including research development, testing, and evaluation, designed to develop or contribute to generalizable knowledge.” (45 C.F.R. 46.102(l)). The definition of research on human subjects is also action oriented. It involves a living individual, about whom an investigator obtains information or biospecimens through intervention or interaction with the individual, and uses, studies, or analyzes it; or obtains, uses, studies, analyzes, or generates identifiable private information or identifiable biospecimens (45 CFR 46.102 (e)). *Identifiable private information* is private information for which the identity of the subject is or may readily be ascertained by the investigator. (45 CFR 46.102(e) (5)). As a consequence, research that neither interacts with the human subject (i.e., does not collect the data) nor uses data with identifiable personal information (i.e., de-identified data) does not fall under the Common Rule (Koch and Todd, 2018). Thus, secondary research on not individually identifiable data that has been obtained, for example, from a public database likely does not fall under the Common Rule. It may neither need IRB approval nor consent (Meyer, 2020). The Common Rule is not clear about the standards for what counts as identifiable personal information and acknowledges the risk that such information could be generated (e.g., by re-identification of non-identifiable data or by merging of information from different sources like coding lists). It therefore implements a regular process of re-examining the definition of identifiable data. The Common Rule suggests that “*broad consent*” should be collected from the participants if identifiable data will be stored, maintained, or processed in secondary research. However, there are also several conditions under which the requirement to obtain consent are waived for research on subjects performed in covered institutions (Koch and Todd, 2018). The control of adherence to the Common Rule of covered research is done by the Office for Human Research Protections (OHPR). Enforcement measures can range from termination of the research, including termination of funding, to the exclusion of the investigator from federal funding. However, the Common Rule does not implement options for legal action for human participants, e.g., in case of privacy breaches or insufficient/inaccurate informed consent (Kulynych, 2007).

The Health Insurance Portability and Accountability Act (*HIPAA*) covers protected health information (PHI) collected by covered entities and their business associates. PHI means *individually identifiable health information* (45 CFR 160.103). This can include neuroimaging, genetic and other health related data. Covered entities can be hospitals (and their neuroimaging units), healthcare providers etc. Under HIPAA *research* is defined as “a systematic investigation, including research development, testing, and evaluation, designed to develop or contribute to generalizable knowledge” (45 CFR 160.501). This definition differs from the Common Rule as it does not require interaction with the participants and therefore secondary data use is not automatically out of the reach of HIPAA. Data protection is implemented by a privacy and a security rule. The latter comprises storing and handling of data while the former defines limits of data sharing and rights of individuals. PHI can be shared with business associates under a contract ensuring adherence to the HIPAA rules. HIPAA requires *written informed consent for data sharing* (Kulynych, 2007). In principle identifiable neuroimaging data could be shared if waiver was granted by an IRB on the basis that the research cannot be performed with de-identified data (Kulynych, 2007; Spector-Bagdady, 2021). However, de-identified neuroimaging data can be publicly shared (disclosed in HIPAA terminology). In contrast to the Common Rule HIPAA provides a set of *approaches to de-identify data*, of which at least one must be implemented (45 CFR 164.514). In concordance with GDPR it requires that “the risk is very small that the information could be used, alone or in combination with other reasonably available information, by an anticipated recipient to identify the individual who is a subject of the information”. The Expert Method requires some expert (e.g., a statistician) to confirm that the risk of identification is low. Alternatively, the Safe Harbor Method requires that faces, biometric, and a list of 16 other identifiers^15^ are removed from the data. A code can be assigned for de-identification that allows a restricted number of persons, with access to the code, re-identification. This is similar to pseudonymization under GDPR. HIPAA also requires sparseness regarding people with access to PHI and the amount of information released. It grants the *release of de-identified PHI under a Data Use Agreement* (DUA) and defines a minimal set of requirements that must be included in these DUAs, such as the prohibition of re-identification. Moreover, participants have the right to access the stored data, to correct it, and the right to restrict the uses and the disclosure (sharing) of the data (Wolf and Evans, 2018). Thus, individuals may have access to raw data and interpreted results. This is in stark contrast to the Common Rule but similar to GDPR. Another important difference to the Common Rule is that individuals have the right to complain to the covered entity and the Secretary if HIPAA rules are violated and the Department of Health and Human Services (HHS) can sanction non-adherence with a civil monetary penalty.

Additional or other regulations hold for research with a different scope. For example, human data collected in NIH funded research fall under a Certificate of Confidentiality policy and FDA regulations apply if human data was collected in the context of medical device or drug development/testing (see e.g., regulation of the test kits for direct-to-consumer genetic testing, Spector-Bagdady, 2021). This multitude of regulations is not only a burden for researchers and human research participants. They also pose a problem for data scientist who want to make use of the data and become even more virulent when the neuroimaging data is augmented by meta- or other data. Rosati (2022) points out that the scopes and concepts of the definitions of de-identified data differ among the Common Rule, HIPAA, and the NIH Data Management and Sharing policy. As a consequence, the same data can be analyzed under different regulatory regimes depending on who analyses them, for what purpose and whether they are de-identified or identifiable.

In sum, a host of regulations exists in the US which cover different institutions and types of research. Despite that, the protection of data from humans voluntarily donating their data for research appears relatively weak. Even the fallback option “Common Rule” does not cover all research uncovered by other regulations. As a simple example the Common Rule does not apply to citizen scientists when they obtain human data (Meyer, 2020). The regulations create space for a field of unregulated research on human subjects and data processing/brokering gained in such research, e.g., privately funded research (Meyer, 2020). Also, research on publicly shared data obtained from open repositories often neither needs ethical review nor consent. Note that the GDPR would still cover secondary data use (e.g., downloaded from a database) and pseudonymized (de-identified) data. The current combination of weak protection of research participants by federal law and case law which favors data collection and access over participants’ autonomy (Kulynych, 2007; Price et al., 2019; Spector-Bagdady, 2021) triggered the development of new data privacy laws like the California Consumer Privacy Act (CCPA), which is strongly oriented along the GDPR. Although CCPA explicitly excludes data regulated under HIPAA, this may be the starting point for a more principled regulation with a wider scope that closes gaps left by existing regulations (Price et al., 2019). On the federal level there is now the American Data Privacy and Protection Act (ADPPA)^16^ in the legislative process, that will largely pre-empt state laws if it comes into force.

#### 2.1.3 China

China’s data protection has been suggested to implement a third way between EU’s GDPR, which implements a basic right for protection of personal information and control by the individual data subject with extraterritorial reach, and the decentralized, application field and data processor oriented regulations issued by different authorities in the US (Pernot-Leplay, 2020). China builds on a *hierarchy of laws* of which the higher level ones, the Cyber Security Law (CSL), the Data Security Law (DSL), the Personal Information Protection Law (PIPL), and the Civil Code of the People’s Republic of China (CCC), constitute a normative, systematic, and complete personal information framework that is supposed to guide regulations released by domain specific institutions (Pernot-Leplay, 2020; Wang et al., 2022). This is reminiscent of the EU approach. The *“lower level” regulations* are then supposed to provide the framework for the handling of data by specific actors in specific fields. This is reminiscent of the situation in the US, where regulations are flexibly defined within certain domains and are only valid there.

The CSL was enacted 2016, 5 years before PIPL, DSL, and CCC which were enacted in 2021. The CSL and the DSL focus on the protection of national security and public interest, while the PIPL and CCC (Art. 1034–1039) focus on the protection of *personal information*. The CSL and DSL implement the principle of *data sovereignty of China*, by giving the state control of over the data acquired on the mainland of China. The DSL categorizes data in the three groups of national core data, important data, and general data where national core data can be subject to cross border protection if they are relevant for national security or public interest (Creemers, 2022; S. Li and Kit, 2021).

The PIPL and the CCC (Art. 1034–1039) protect personal information rights and interests of natural persons and seek to promote the appropriate use of personal information (PIPL Art. 1; Cheng, 2022, see Presentation 1 in Supplementary Material for original Chinese version of this publication and Table 1 for a translation into English of the important sections). They distinguish private and non-private information; sensitive and non-sensitive personal information. PIPL is superficially reminiscent of the GDPR but has important differences as it puts more emphasis on the governance model under the principle of national sovereignty. PIPL considers it the state´s task to safeguard personal data at the national and international level and delegates protection to other laws, administrative regulations, and infrastructure programs.

In Article 4 PIPL^17^ defines *personal information* as information related to identified or identifiable natural persons as opposed to anonymous information. *Anonymous* information is defined in a very strict sense as “impossible to distinguish specific natural persons and impossible to restore” (PIPL Art. 73 (4)). Data handlers must *de-identify* personal information to ensure it is impossible to identify specific natural persons without the support of additional information (PIPL Art. 73 (3)). This is similar to the concept of pseudonymization in the GDPR or de-identification under HIPAA.

PIPL does not distinguish data controller from data user and subsumes the concepts under the term data handler. The *data handler* is responsible for the security of the personal information they handle (PIPL Art. 9). Articles 51–59 define their duties and Articles 66–71 define legal punishments for violations of the laws and regulations on personal information handling, including monetary penalties. Interestingly, they also define penalties for the responsible person(s) for failures of state organs to protect personal information.

PIPL requires *informed consent* from the data subject for personal information handling (PIPL Art. 13) but provides many exceptions, including other laws and regulations. The consent must be detailed (e.g., purposes of data handling, transfer abroad etc.) and must be obtained again if new purposes of data handling are intended (PIPL Art. 14) but it can be withdrawn by the data subject (PIPL Art. 15). Interestingly, at the level of PIPL there is no mention of independent review boards in the sense of IRBs.

PIPL additionally defines *sensitive* personal information which includes, among others, biometric characteristics and medical health data (PIPL Art. 28 (1)). The handling of sensitive personal information should comply with the principle of “specific purpose” plus “separate consent” (Wang, 2022, see Presentation 2 in Supplementary Material for original Chinese version of this publication and Table 1 for a translation into English of the important sections). Firstly, the handling of sensitive personal information must be for a specific purpose and with sufficient necessity, as well as with strict safeguards (PIPL Art 28 (2)). Secondly, the *separate consent* must be obtained for handling sensitive information (PIPL Art. 29). However, the concept of a „specific purpose” is indistinct. In addition, many details of the practical implementation of handling sensitive information is delegated to other laws and regulations.

Article 36 of PIPL requires personal information handled by state organs to be *stored on mainland China*. This likely includes the majority of neuroimaging, genetic and other research data. Articles 38–43 regulate sharing of personal information across borders. They require justifications for *sharing abroad*, security assessments, standard contracts, notification of the data subjects, and put the burden to control adherence of the foreign receiving party to the regulations onto the data handler. In addition, Article 53 requires from the extraterritorial data handler the appointment of a *representative on China mainland* who must be reported to the relevant departments. This could mean that a collaborator from China is necessary when human research data from there are processed abroad. Articles 44–50 provide data subjects the right to require data handlers to provide, correct, transfer, or delete their data. Articles 60–65 define departments responsible for the oversight over the personal information protection, putting the Cybersecurity and Information Department at the top of the hierarchy. Here it is also stated that everyone has the right to complain about unlawful personal information handling activities.

PIPL is a relatively new law and the future will show which effects it has on sharing of neuroimaging data. Even before PIPL came into effect, several constraints on international research data exchange (e.g., the access of foreign researchers to genetic data collected on mainland China) were implemented in laws and regulations for state reasons. In March 2018, the State Council issued the “Measures for the Management of Scientific Data”, or short “*The Measures*”^18^. The Measures are binding for research institutions. They state that a scientific data archive system should be established and that government-funded scientific data should be submitted to this data center (The Measures Art. 12 & 13). For data produced in government funded research the Ministry of Science and Technology (MOST) can decide whether data can be shared or not. Among the criteria for restricting sharing are whether the scientific data contain personal information or concern national security. The adherence to the privacy laws is supposed to be implemented at the level of the data center policies which are currently evolving (Li et al., 2022). The complex and domain specific regulatory framework and its consequences for international sharing has been mostly analyzed in the field of *human genetic data* where China has considerable data resources. Chen and Song (2018) provide an overview of the laws and regulations and conclude that while data privacy plays a role in the regulation of data transfers, the national interest and security became a main reason for their protection by restricting their processing to the China mainland and requiring researchers from abroad to collaborate with a Chinese researcher or institution if they want to process genetic data collected in China (Chen and Song, 2018; Mallapaty, 2022). Sharing of *neuroimaging* data may be less affected by national interests as long as they are not considered health data. Regulations like safety assessment, data use agreement, data protection impact assessments, and consent for transfer may apply only from a certain size of the data sets upwards (PIPL Art. 52, Mallapaty, 2022). Moreover, the MOST has recently released a very general set of ethical norms for the use of AI in China which also covers the use and protection of personal information (Dixon, 2022).

In sum PIPL has superficial similarities to the GDPR in that it provides data subjects similar protection mechanisms (personal data, special data, requirement of consent for processing, right to withdraw consent, right to obtain information, correction/deletion of data etc.) and mechanisms to enforce their rights. These protection rights are sometimes even stronger than in the GDPR. However, it is formulated in a very general way and relies on domain specific regulations implemented by the respective authorities, similar to the data protection regulations in the US. With the additional CSL and DSL it implements mechanisms that allow state authorities to control the transfer and processing of data collected in the mainland of China to researchers abroad, thereby establishing mechanisms to enforce data sovereignty of the state. These export restrictions already have some effects in the field of human genetics. As PIPL and DSL are relatively new laws and the specific regulations are currently emerging it remains to be seen what impact they will have on the exchange of neuroimaging data.

#### 2.1.4 Summary

The review of the three systems must remain incomplete in breadth as well as in depth. However, it highlights some convergences and differences between the regulations in three geographic and cultural regions that can be considered as among the top scientific data generators and their regulations span a spectrum in which most of the currently emerging data privacy regulations may be contained. Convergent among the regulations is that they all have regulations to protect private information and sensitive data. They all require explicit informed consent for the acquisition and sharing of data and, in general, require that the data is at least de-identified/pseudonymized or anonymized before data processing and sharing. They all suggest or require some form of contractual agreement between the data supplier and the data receiver to ensure that the data is processed in concordance with the regulations of the country in which they were acquired. While there is agreement in the subject of protection and some general means for protection of human research data, there is a great diversity in the way how the regulations are implemented and in their reach of protection. While the EU GDPR puts the protection of the individual at the center and seeks to balance it with the interest of science, the US regulations tend to favor the accessibility of data for science and economy over privacy. Laws and regulations in China emphasize both the protection of the individual as well as state interest and data sovereignty. Moreover, the regulations differ considerably with respect to accountability, liability, and sanctioning with the most lenient regulations in the US and potentially the most comprehensive definitions of responsibilities in China’s laws. Importantly care should be taken when matching terminology across jurisdictions (Eke et al., 2021). The same terms may have somewhat different meanings and some functional roles that may be distinguished in one context (e.g., data controller and data processors in GDPR) but lumped into one role in another (data handler in PIPL). It is, however, encouraging that there appears to be sufficient overlap that a limited set of measures could allow neuroimaging data sharing in a way that is compatible with all three sets of regulations for privacy protection. Table 2 provides an overview of such measures for the three jurisdictions. However, this should be further analyzed and corresponding procedures should be developed.

**TABLE 2:** Some points researchers need to consider when sharing data or using shared data.

EU	United States	China
IRB, approval of lawfulness of processing, pseudonymization (anonymized data not covered by DPR). Specific consent important for legal sharing.	Under HIPAA: IRB, de-identification or anonymization (e.g., Safe Harbor Method), consent for sharing and/or DUA, depending on type of data.	De-identification. Very high standard for anonymization. Detailed consent for all forms of processing.
Lawful sharing possible within EU an states with adequacy decision. DUA to restrict processing purposes of data recipient outside EU.	No restriction for sharing into different countries.	Complex procedure for sharing outside mainland China. May require collaborator in China or be impossible depending on data classification.

### 2.2 The (meta)data description challenge

Even when legal regulations are met and datasets are publicly shared, it is not guaranteed that the information in them is accessible and useful for a data processor. The FAIR principles (Wilkinson et al., 2016) state some general requirements of how scientific data should be handled and documented to make them useful for others. The acronym FAIR stands for Findable, Accessible, Interoperable, and Reusable. Findability means that the data is either aggregated in a way (e.g., on a server) that the user knows where to look for them or that they are equipped with descriptive metadata such that some sort of search engine can retrieve their location. In addition, data should have a persistent identifier, such as a digital object identifier (doi), to assure findability over a long time period. Accessibility refers to the ability of a human or a computer to either retrieve the data from their storage location or to run them through an analysis pipeline on a remote server without retrieving them. Interoperability means that data should integrate into different data analysis ecosystems as well as the integration of data with other data. In particular with big data, interoperability is necessary to use the data on computers without or with minimal human interaction. Reusability aims at efficient data use which is of particular importance for data that are rare or expensive to produce. It means that data should not only be useful for the purpose they were originally collected for (Bigdely-Shamlo et al., 2020; Niso et al., 2022).

To match these requirements, research data need to be organized according to some standard. Using a standardized data structure alone, however does not suffice to ensure that shared data become findable, interoperable, or reusable, for example, in large-scale meta studies. Proper description of the data is another requirement. This has been pointed out, among others, by the European Commission expert group for FAIR data. The expert group recommended comprehensive documentation of research products, such as experimental data or analysis pipelines, through metadata (European Commission and Directorate-General for Research and Innovation, 2018). Ideally, these metadata are based on standard vocabularies or ontologies, which add semantics to the terms of the vocabulary.

The domains for metadata range from descriptions of the human participants, the experiment, the nature of the experimental data, additional tests and surveys, to consent and usage restrictions. Even though, many publicly shared datasets contain some metadata, these are likely not descriptive enough to effectively re-use them and working with such data can be error-prone and tedious (Niso et al., 2022). Additionally, metadata are often described in idiosyncratic terminology of the researchers, who share the dataset, making them hard to interpret for (other) humans and impossible for machines. This severely restricts findability, interoperability, and reusability. The latter particularly in the context of big data research efforts. One way to cope with this problem is to define vocabularies or even ontologies, which can then be used to annotate the data in a standardized manner. For example, a neuroimaging dataset with standardized event annotation can be re-used for purposes it was not originally collected for (Bigdely-Shamlo et al., 2020; Niso et al., 2022), simply because the experiment may include events, that were unrelated to the original research question but necessary for the structure of the experiment (e.g., button press events that require motor responses which might not have been in the scope of the original study). Ideally, if augmented by rich metadata, complex datasets can be used in many studies with different purposes (e.g., United Kingdom Biobank^19^, Study Forrest^20^)

Recently, the neuroimaging community elaborated open standards for data storage yielding common structural organizations of raw datasets from different modalities (Teeters et al., 2015; Gorgolewski et al., 2016; Niso et al., 2018; Pernet et al., 2019). The most commonly used is the Brain Imaging Data Structure (BIDS^21^, Gorgolewski et al., 2016). Importantly, many neuroimaging data analysis tools have adopted the standard and interoperate on it to some degree. However, the standardization is still not comprehensive enough to guarantee the full FAIRification of datasets including derivatives. Moreover, other scientific communities may have different standards that may be less developed or lack standards at all. The reasons for that can be manifold, including but not restricted to the lack of a culture supporting sharing, the ubiquitous use of closed commercial systems, or particularly strong data protection constraints due to commercial interests, as in industry or in the health domain. Since we cannot cover the wide range of data standards in this paper, we focus on BIDS as a showcase for structured data storage enriched with some metadata.

#### 2.2.1 BIDS

BIDS is a community driven project to abstract and standardize the representation of neuroimaging data. Essentially it breaks down to a hierarchical directory structure with specific data-file and folder naming conventions plus some standardized metadata for the description of the image acquisition and the event annotations of the experiment (given that the experiment deploys a task-based structure). Importantly, BIDS is not only defined as a human readable directory hierarchy but also as a computer interoperable schema, which allows for more flexibility, is less error-prone with respect to maintenance of the standard, and facilitates the usage of automated processing pipelines on BIDS datasets. Moreover, the metadata and some of the data (e.g., timing of events) are also human readable, which eases the understanding of the dataset. Such a unifying data structure carries the potential to make neuroscientific research more transparent and encourages data sharing between researchers and labs.

These advantages of BIDS only apply if the data structure is widely accepted and used. For this reason many experts from the neuroimaging community were consulted during the development of BIDS to create a data format which is intuitive and easy to use while being able to handle a variety of experimental data, e.g., from different modalities such as fMRI (Gorgolewski et al., 2016), EEG (Pernet et al., 2019), MEG (Niso et al., 2018), behavioral data, and many more. It can thus be used for most experiments and even across imaging techniques for the standardized storage of multimodal datasets. Since BIDS is a rather young development and open source, it is constantly evolving to describe more aspects of the data acquisition and the respective analyses applied.

BIDS defines some basic data acquisition related metadata and strongly recommends to include them in every dataset. Additionally, BIDS requires that metadata are stored in the Java Script Object Notation (JSON), an open and text-based file format consisting of attribute-value pairs that are both human and machine readable. Even though these JSON files are not mandatory according to the BIDS specification, they are most often included in (publicly shared) BIDS datasets, simply because the tools that convert datasets from the vendor specific format to BIDS extract them from the former and write them to the JSON-files of the latter. These conversion tools are currently best developed in the MRI domain, e.g., HeuDiConv (Halchenko Y. et al., 2021) and dcm2bids^22^, but there are ongoing community efforts to facilitate the conversion to BIDS for other modalities, such as MEEG (MNE-BIDS^23^, Appelhoff et al., 2019). However, these basic metadata defined in BIDS do not suffice for exhaustive description of the raw data nor for the description of analyses employed to obtain data derivatives, e.g., results of an analysis. One of the reasons is that BIDS defines a framework for several data acquisitions modalities, all of which require domain specific metadata. Additionally, different fields of research may require different metadata which again adds complexity to the task of developing an exhaustive, overarching and modality agnostic metadata standard within BIDS.

#### 2.2.2 HED tags and the neuroimaging data model (NIDM)

In the neuroimaging domain the Hierarchical Event Descriptor standard (HED^24^, Bigdely-Shamlo et al., 2016; Robbins et al., 2021) is an infrastructure which defines rules for controlled and hierarchically organized vocabularies. Terms from these vocabularies can then be used to describe the nature and time course of an experiment, that was performed while brain data was recorded, by tagging the data with keywords while assuring findability of these tags during downstream analyses. The HED base schema defines a hierarchical vocabulary for the description of basic stimuli, responses, tasks and experimental conditions. However, more specialized or domain specific vocabularies/schemas can be added to the standard as long as they adhere to the rules for schemata defined by HED. One example is the SCORE vocabulary for clinical EEG annotation (Beniczky et al., 2013, 2017), which has been converted to an HED schema and is currently under community review. Moreover, existing vocabularies can be extended to cover a wider range of applications or use cases. HED was developed in a community effort, recently fully integrated into the BIDS ecosystem and since the release of BIDS 1.8.0. tagging data with terms from, multiple vocabularies is accepted^25^.While far from being able to completely annotate all research products, like analysis pipelines, the HED vocabularies are an important ingredient to make data sets machine actionable and reduce ambiguity for human researchers. Moreover, tagging your data with these standardized HED-tags allows for better collation of separately recorded datasets.

The Neuroimaging Data Model (NIDM^26^, Keator et al., 2013; Maumet et al., 2016) complements HED by providing additional functionality, such as the description of analysis workflows and results (though currently limited to MRI-data). Importantly it provides methods to describe the provenance of research products, i.e., the way they were generated. Provenance documentation is expected to increase reproducibility and to improve the usefulness of sharing analysis methods. NIDM employs different components to model different aspects of the data: NIDM Experiment for capturing and annotating experimental metadata (similar scope as HED), NIDM Workflow for the standardized description of analysis workflows, and NIDM Results (Maumet et al., 2016) for standardized description of results including provenance information. It should be noted, however, that these components are at different stages of development, with the NIDM Results being the most sophisticated. NIDM is a spin off from the US Brain Initiative and is based on Semantic Web technology. It is mainly based on the PROV (provenance) vocabulary (Moreau et al., 2015). However, it also incorporates terms from several other vocabularies or ontologies such as the Dublin Core^27^ for file description and the STATisical Ontology (STATO)^28^ for the annotation of statistical methods like General Linear Models. Additionally, the NIDM developers have started to map terms/study variables, commonly used in openly shared datasets, to concepts from existing ontologies/vocabularies, such as the Cognitive Atlas (Poldrack et al., 2011) or the InterLex information resource. This initiative is called the NIDM-Terms^29^ and community efforts to expand this ontology are welcome.

In practice an immense amount of data and metadata standards exist even within such a small research field as neuroscience. Many of those standards are very narrow in their range of application, lack community/institutional support, and are potentially overlapping. This could lead to suboptimal use of human as well as financial resources. In an effort to integrate the different standardization approaches, the open Metadata Initiative for Neuroscience Data Structures (openMINDS^30^), which emerged from the EU Human Brain Project, aims to collect and integrate metadata standards into an overarching ontology to connect terminologies used in various fields of neuroscience. In addition, they also collect frequently used brain atlases and common coordinate spaces for neuroimaging data. Similar to NIDM, the openMINDS project is subdivided into several modules, which differ with respect to their level of development.

#### 2.2.3 Metadata and privacy protection

Metadata annotations and privacy protection in legal frameworks may appear as two different challenges to the same problem, the lack of useful openly shared data. However, they are potentially connected. Data which is equipped with rich metadata is more likely to be de-identified and hence the developers of vocabularies or metadatmodels need to be cautious when including terms which could be mapped to identifiable information. More general, there is a trade-off between data which is perfectly described by metadata and minimizing the risk of re-identification. Additionally, the safeguards that need to be implemented and the metadata that need to be removed or “filtered” can vary depending on the legal regulations that apply to the data. However, little is currently known to what extend comprehensive sets of metadata may impact privacy protection in practice, how metadata could be exploited by future AI techniques, and how safety assessments would change with increasing volumes of findable, openly accessible, and properly annotated data.

## 3 Practical solutions

So far, we have covered important factors that may have a negative impact on useful data sharing, i.e., lawful sharing of data, that can be easily understood and interpreted. We also covered the benefits for the individual researcher and society. In this last section we want to introduce some tools, practices and initiatives that support the individual researcher in reducing the additional effort/labor associated with data sharing. Some of these may be specific to data from human neuroimaging but others might be more general, applying to a wide range of data types from different fields.

### 3.1 Consent and anonymization

A recent survey on open science practices in the functional neuroimaging community revealed that 41% of the researchers did not share their data due to the fact that their consent forms excluded the option for data sharing (Paret et al., 2022). Hence, researchers who plan to share data should take care to design the consent form in a way that data can be shared on a lawful basis or include a consent form that was specifically designed for that purpose. Obtaining explicit consent is one central building block for lawful data sharing. However, researchers should be aware that the informed consent to participate in the experiment does not entail consent to sharing the data with others. The explicit consent to sharing the data can be integrated into the informed consent form, though. This must be done in a way, such that the data subject clearly understands that their data might be shared with the research community in a pseudonymized form. Moreover, data subjects should understand the researcher’s role in mitigating the risk of a privacy breach through re-identification. In order to simplify that step, the Open Brain Consent (OBC) working group (Bannier et al., 2021) provides template consent forms in many languages on their website^31^. They are designed to meet the requirements for explicit consent under the GDPR. Table 2 lists some points to consider for lawful data sharing in different jurisdictions. It should be noted here however, that the final decision whether obtaining informed consent for public sharing of pseudonymised data is in the hands of the data protection office of the research facility, and in practice their assessment may vary between institutions.

Besides obtaining consent, anonymization, de-identification or pseudonymization (in case anonymization is not possible) of the data are required in any of the legal frameworks covered here. There are numerous techniques for anonymization, de-identification and pseudonymization. If unsure which technique to use, the European Data Protection Working Party has issued an opinion on anonymization techniques^32^ in 2014, highlighting benefits and potential pitfalls of several anonymization approaches including differential privacy, randomization, noise addition, permutation, generalization, and L-diversity/T-closeness. Additionally, several free and open-source tools exist to apply these techniques. For example, the ARX anonymization tool^33^ (Prasser et al., 2014) provides functionality to anonymize data and additionally analyze the risk of re-identification for the chosen anonymization/de-identification technique. These general tools are useful for metadata. Neuroimaging data are more complex, since not only metadata need to be curated to achieve anonymization. In the case of fMRI all facial features need to be eliminated, a process called defacing. The OBC working group (Bannier et al., 2021) again provides links to some useful tools on their website^34^, e.g., tools for sanitizing the DICOM header and tools for defacing. For example, BIDSonym^35^ (Herholz et al., 2021) provides an interface for BIDS data which allows defacing using different techniques.

### 3.2 Data user agreements and databases

Data user agreements (DUA) are one option to bind the data processor (entity that receives the data) to some set of predefined conditions when accessing the shared data. This is particularly important when they belong to the category of sensitive data. DUAs have become a prominent way to mitigate the misuse of data and are applicable in different jurisdictions. A DUA is a contract between the data controller and an external entity or the person seeking to access the data. It defines a set of rules around the shared data. With such agreements a data controller can control with whom or for what purposes they want to share the data. For example, data can be shared under the constraint that no re-identification will be attempted, or for scientific research purposes only, thereby excluding the use of the shared data for economic purposes. DUA’s are endorsed by the European government and are a step towards fulfilling the principle of privacy by design, as required by the GDPR. An exemplary template of a DUA is provided on the OBC’s webpage^36^.

Providers of public data platforms or repositories need to implement a mechanism to handle and store such (digital) contracts. Moreover, these platforms need some kind of access control and identification mechanisms, since DUAs are legally binding contracts. Unfortunately, many well-known public and open neuroimaging data repositories, e.g., OpenNeuro (Markiewicz et al., 2021), Distributed Archives for Neurophysiology Data (DANDI)^37^, and the International Data-Sharing Initiative (INDI)^38^ have hitherto not or only partially implemented infrastructure for DUAs or access control mechanisms. While this might be sufficient to share some data acquired in the US, it may not suffice for data acquired under HIPAA, the GDPR and Chinese laws and regulations. However, there are also several data platforms that allow lawful sharing under the GDPR with the required safeguards. For example, Ebrains^39^ provides a platform for sharing several kinds of data. There, uploading data is only possible if certain technical and organizational measures for safeguarding the individual’s right to privacy are met. There the data needs to be de-identified, anonymized or pseudonymized and is additionally safeguarded (encrypted) *via* the Human Data Gateway. Moreover, the users who want to access data need to comply to a given set of conditions, one of which is the acceptance of an additional DUA. Another example is the Open MEG Archive (OMEGA, Niso et al., 2016). This is a data repository specialized on MEG data. It implements a controlled access mechanism (institutional credentials are necessary to create an account) and requires signing a DUA before data access. A list of some online data repositories with information on the safeguards that these databases have implemented can be found in Eke et al. (2021).

### 3.3 User-to-data

The concept of user-to-data describes an alternative approach to data custodianship to avoid legal issues revolving around shared data. The idea behind this concept is that data does not need to change its location (the server or computer it is stored on) to be useful to many people. Instead, users can be “moved” to the data by giving them means to work on the data and run analyses on them without having full access rights, e.g., researchers can not see or copy the data. Consequently, this requires the host websites to provide some kind of interface for working with the data on their servers. One example of this approach is brainlife^40^. This platform also provides sufficient computing power to run analyses, test algorithms or to benchmark software and has streamlined access to data from various open databases. However, brainlife does not entirely exclude the option to download data processed on their servers. A Data Safe Haven provides a secure environment for the analysis of sensitive data with appropriate technical and informational governance mechanisms. Data Safe Havens have been developed at several institutions and universities, such as the UCL^41^, or the university of Hull^42^. The Turing Data Safe Haven^43^ is a resource that comprises general information on Data Safe Havens as well as scripts and templates to set-up and maintain such secure environments. Moreover and very recently, several initiatives have emerged targeting the facilitation of setting up privacy preserving frameworks for the analysis of sensitive data, such as Vantage6^44^ or OpenMined^45^. Vantage6 is an open source infrastructure for privacy preserving analysis. It provides functionality for servers, which allow setting up “data stations” which securely store the data. Algorithms can be delivered to these “data stations” and results will be sent back to the user. OpenMined is a movement, which is composed of three programs: the build, the educate, and the impact program. The build program is about developing tools to help setting up privacy preserving data analysis environments. This is similar to Vantage6, though with a strong focus on running AI methods on the data. The educate program clearly is about education of remote data science, especially since this is a comparatively new field of data science. They provide several courses to learn more about remote data science and working with their PySyft^46^ library. The impact program is about showing that the developed tools work by teaming up with partners from public and private organizations to test the generalizability and usability of them in a variety of use cases. The user-to-data approach seems to be promising to enable data access for many people with minimal legal constraints, however, it needs to be considered that limited compute and storage capacities might be the bottleneck of this approach. Additionally, the maintenance of the infrastructure is complex and expensive. Smart data management tools, such as Datalad (Halchenko Y. O. et al., 2021), can promise some relief to the resource problem by employing a decentralized structure (Hanke et al., 2021), e.g., servers for databases need not be at the same physical location. Detailed information on Datalad, e.g., its usage and range of application, can be found in the Datalad Handbook (Wagner et al., 2021). Finally, the speed of technical development might also mitigate the issues with resources.

### 3.4 Tools for data and (meta)data handling

Making a dataset useful for other researchers can be costly. Data and metadata standards support this task. Fortunately, tools exist that help implementing these standards in everyday scientific practice. They support data transformation, metadata annotation, and data handling in general. This can include software for the conversion into a given data storage standard or file format, software for data management, parsers for specific file formats, tools to filter the dataset for specific metadata, ideally with many options for queries, tools for validation of adherence to a given standard, and tools for metadata extraction or editing of metadata files.

In Section 2.2.1 we mentioned some tools that help with the conversion of rawdata into BIDS, covering several modalities and programming languages. In addition, the BIDS community offers a web-based tool for the validation process. For interaction with the BIDS converted data stored locally, BIDS-Matlab (Gau et al., 2022) and PyBIDS/ancpBIDS (Yarkoni et al., 2019) are commonly used tools. Both allow for complex queries on the data, hence many filtering options and provide an API for their integration into custom workflows or pipelines. Moreover, the DataLad (Halchenko Y. et al., 2021) family provides useful functionality for decentralized data management (i.e., data that is stored on several servers or repositories), while additionally tracking the provenance of all files in a dataset. Extensions to DataLad target more specific aspects of data handling. For example, MetaLad^47^ is a tool which is specifically designed to facilitate the handling of metadata. It can deal with various file formats and provides useful functionality, such as filtering existing metadata, e.g., for specific keys, or the extraction and aggregation of metadata. On top of that, DataCat^48^ is another DataLad extension, which eases user interaction with the metadata by providing browser-based and easy-to-navigate-through metadata catalogues, i.e., a user interface which facilitates metadata inspection and handling. Note, that DataCat is still under development and no stable version exists yet.

Additional tools are available for working with the metadata standards mentioned in Section 2.2.2. The NIDM team has developed a python-based command line tool (PyNIDM^49^) and an additional web application which allow the user to convert BIDS data into NIDM files, interactively map terms (e.g., study variables from a tabular sidecar file) to concepts in existing ontologies/vocabularies or to define new terms. These tools also allow the creation of JSON-formatted data dictionaries, e.g., with provenance information, which are then stored as sidecar files alongside the data. Additionally, the developers of HED provide several online tools^50^. They include tools for validation, summarization and generation of BIDS compatible events-files, tools for the generation, validation, transformation, extraction and merging of respective JSON sidecar files, which are designed to semantically describe the columns of the events-files. Moreover, HED offers a tool to validate and convert new schemas or extensions to existing schemas (vocabularies). All of these tools are intuitive and easy to use and provide a self-explaining browser-based user interface and unlike command line tools, the HED online tools do not require any prior experience in programming or any operation system specific knowledge since they are browser based. Technically, this should also enable the user to make use of these tools on mobile devices, such as tablets.

The scope of this paper does not allow for an exhaustive list of tools and practices for open neuroimaging. Therefore, we refer the interested reader to Niso et al. (2022) and, in particular, the table in the supplementary material, for a more detailed overview of available open science tools and practices, that support transparent and reproducible research at every stage of the research cycle.

## 4 Conclusion

Despite the manifold benefits of shared data for individual researchers, the scientific community and society, only a small fraction of data generated in life sciences is made openly available (Houtkoop et al., 2018). Moreover, the data, that is openly shared, is often of limited use because it is not saved in a standardized way and/or insufficiently described. This renders them hardly understandable for humans and prevents automated computer interoperability. Here, we cover the two important factors contributing to these problems: insecurities around the lawfulness of data sharing as well as missing metadata and standardized data organization. Many individual researchers withhold their data because they lack knowledge about options for sharing and are afraid of legal implications of privacy protection laws (Eke et al., 2022). In order to shed light on options and constraints for sharing human neuroimaging and comparable human data, we provided an overview of relevant legal frameworks in the three geographic regions with the largest data resources, provided an accessible tabular overview, provided a concise overview of points to consider when planning to share data, and introduced platforms and procedures that support lawful human research data sharing. In order to ease the burden of standardizing data organization and annotation we introduced initiatives, that develop standardized data structures and vocabularies for the description of neuroimaging data. Additionally, we provided an overview of free, community developed, and open source tools and databases that simplify the construction and reproduction of analysis pipelines by integrating standards and practices, covered here, into the research workflow. The mentioned tools/initiatives/practices can drastically reduce the over-head for FAIR and lawful data sharing for the individual researcher, increase the efficiency of data handling, and increase the reusability of the data and thereby their value for the individual researcher, the scientific community, and society.

At a first glance, the three legal frameworks covered here appear very different and they are, when scrutinizing details like the definitions of terminologies, their reach of protection and the implemented mechanisms for sanctioning. However, at a practical level, there is quite some overlap among the requirements for research data sharing: A combination of IRB, detailed explicit consent, and pseudonymization is at the core of all regulations and established practice in the majority of (neuroimaging) labs handling human data. Additionally, DUAs help with sharing data requiring special protection. However, there are several domains, in which further improvements are desirable. In the foreseeable future, DUAs and user-to-data platforms may play a bigger role if the volume of internationally shard data increases. More and better tools are required to support this development as only few and often local user-to-data platforms exist and the handling of DUAs is still in its infancy and not really useful in AI applications aiming to include datasets from distributed sources in addition to, or instead of, centralized large databanks. Moreover, the assessment of risk for re-identification seems underdeveloped for neuroimaging data compared to some common metadata, for which risk-assement procedures and tools already exist. However, the interactions between neuroimaging and metadata in risk assessment seems unexplored although such interactions can be expected. At the level of legal regulations, it has been reported that the GDPR serves as a blueprint for many privacy protection laws that are currently developed or updated in countries around the world (Greenleaf, 2022). This trend may support the homogenization of privacy protection laws across jurisdictions and as a consequence allow the development of some generalizable core practices for sharing, although local regulatory idiosyncrasies, that need to be met, will likely continue to exist.

Shared data must meet some requirements to be useful. Among others are adherence to a well-established open data standard that is supported by tools for data conversion, data handling and frequently used analysis tools. Moreover, standardized metadata are necessary to make them understandable. So far only few tools exist to augment the core data with metadata and to process them. Standardization of data storage formats and metadata is core to make a dataset FAIR and useful for humans and machines. Most researchers may have searched for a data reader because the favorite analysis tool cannot processes the format of the desired data. Many may be familiar with the guessing whether “RT” in one dataset may mean the same as “index” in another, and “button press” in a third. Such obstacles can, in principle, be removed when open data standards are used. However, when it comes to choosing a standard the blessing of many options can turn into a burden. Our own approach to the choice problem is to consider a) wide acceptance and adoption in the community, b) the existence of tools that support the application to the data, c) support of the standard by tools used in the analysis workflow or even automation of it, d) sustainability supported by a strong community that continuously develops the standard and respective tools, e) that time to develop idiosyncratic solutions for an individual lab is often wasted and better invested in the support of community developments.

## Footnotes

1
https://www.humanbrainmapping.org/i4a/pages/index.cfm?pageid=1; last accessed: 26.10.22.

2
https://www.incf.org/; last accessed: 26.10.22.

3
https://open-sci.cn/; last accessed: 26.10.22.

4
https://www.genome.gov/human-genome-project; last accessed: 26.10.22.

5 European Data Protection Supervisor. (2020). A Preliminary Opinion on Data Protection and Scientific Research. URL: https://edps.europa.eu/sites/edp/files/publication/20-01-06_opinion_research_en.pdf; last accessed: 25.10.2022.

6 European Data Protection Supervisor. (2020). A Preliminary Opinion on Data Protection and Scientific Research. URL: https://edps.europa.eu/sites/edp/files/publication/20-01-06_opinion_research_en.pdf; last accessed: 25.10.2022.

7
https://digital-strategy.ec.europa.eu/en/policies/privacy-mobile-health-apps; last accessed: 25.10.22.

8 European Data Protection Supervisor. (2020). A Preliminary Opinion on Data Protection and Scientific Research. URL: https://edps.europa.eu/sites/edp/files/publication/20-01-06_opinion_research_en.pdf; last accessed: 25.10.2022.

9
https://ec.europa.eu/info/law/law-topic/data-protection/international-dimension-data-protection/adequacy-decisions_en; last accessed: 24.10.2022.

10
http://data.europa.eu/eli/reg/2022/868/oj; last accessed: 25.10.22.

11
https://health.ec.europa.eu/publications/communication-commission-european-health-data-space-harnessing-power-health-data-people-patients-and_en; last accessed: 21.10.2022.

12 https://www.europarl.europa.eu/RegData/etudes/ATAG/2020/652073/EPRS_ATA(2020)652073_EN.pdf; last accessed: 28.10.2022.

13 European Data Protection Supervisor. (2020). A Preliminary Opinion on Data Protection and Scientific Research. URL: https://edps.europa.eu/sites/edp/files/publication/20-01-06_opinion_research_en.pdf; last accessed: 25.10.2022.

14 National Commission for the Protection of Human Subjects of Biomedical and Behavioral Research. (1979). The Belmont report: Ethical principles and guidelines for the protection of human subjects of research. U.S. Department of Health and Human Services. Retrieved from https://www.hhs.gov/ohrp/regulations-and-policy/belmont-report/read-the-belmont-report/index.html; last accessed: 26.10.22.

15
https://www.law.cornell.edu/cfr/text/45/164.514; last accessed: 01.03.2023.

16
https://www.dataguidance.com/jurisdiction/usa-federal; last accessed: 28.10.22.

17 Creemers R. and Webster G. (2021) Translation: Personal Information Protection Law of the People’s Republic of China–Effective Nov. 1, 2021. Retrieved from https://digichina.stanford.edu/work/translation-personal-information-protection-law-of-the-peoples-republic-of-china-effective-nov-1-2021/; last accessed 24.10.2022.

18
https://www.sciping.com/33787.html; last accessed 24.10.2022.

19
https://www.ukbiobank.ac.uk; last accessed: 26.10.22.

20
https://www.studyforrest.org/; last accessed: 26.10.22.

21
https://bids.neuroimaging.io/; last accessed: 26.10.22.

22
https://unfmontreal.github.io/Dcm2Bids/; last accessed:02.02.2023.

23
https://mne.tools/mne-bids/stable/index.html; last accessed: 30.11.22.

24
https://www.hedtags.org/; last accessed: 25.10.22.

25
https://bids-specification.readthedocs.io/en/stable/appendices/hed.html#hierarchical-event-descriptors; last accessed: 30.11.2022.

26
http://nidm.nidash.org/; last accessed: 25.10.2022.

27
https://www.dublincore.org/specifications/dublin-core/dcmi-terms/; last accessed: 30.11.2022.

28
http://stato-ontology.org/; last accessed: 30.11.2022.

29
https://nidm-terms.github.io/info.html?#about; last accessed: 26.10.2023.

30
https://ebrains.eu/news/new-openminds-metadata-models/; last accessed:25.10.2022.

31
https://open-brain-consent.readthedocs.io/en/stable/index.html; last accessed 22.01.2023.

32
https://ec.europa.eu/justice/article-29/documentation/opinion-recommendation/files/2014/wp216_en.pdf; last accessed: 24.01.2023.

33
https://arx.deidentifier.org/anonymization-tool/; last accessed 22.01.2023.

34
https://open-brain-consent.readthedocs.io/en/stable/index.html; last accessed 22.01.2023.

35
https://github.com/PeerHerholz/BIDSonym; last accessed: 12.02.2023.

36
https://open-brain-consent.readthedocs.io/en/stable/index.html; last accessed: 22.01.2023.

37
https://dandiarchive.org/; last accessed 24.01.2023.

38
http://fcon_1000.projects.nitrc.org/; last accessed: 24.01.2023.

39
https://ebrains.eu/service/share-data; last accessed: 21.10.2022.

40
https://brainlife.io/projects; last accessed:21.10.22.

41
https://www.ucl.ac.uk/isd/services/file-storage-sharing/data-safe-haven-dsh; last accessed: 12.02.2023.

42
https://datasafehaven.hull.ac.uk; last accessed: 12.02.2023.

43
https://alan-turing-institute.github.io/data-safe-haven/develop/overview/index.html; last accessed: 12.02.2023.

44
https://distributedlearning.ai; last accessed: 12.02.2023.

45
https://www.openmined.org; last accessed: 14.02.2023.

46
https://github.com/OpenMined/PySyft; Last accessed: 14.02.2023.

47
https://github.com/datalad/datalad-metalad; last accessed: 03.02.2023.

48
https://github.com/datalad/datalad-catalog; last accessed: 03.02.2023.

49
https://github.com/incf-nidash/PyNIDM; last accessed: 27.01.2023.

50
https://hedtools.ucsd.edu/hed; last accessed: 26.01.2023.
