# Supplementary material for: FAIR human neuroscientific data sharing to advance AI driven research and applications: Legal frameworks and missing metadata standards
Source: Front Genet. 2023 Mar 13;14:1086802. doi: 10.3389/fgene.2023.1086802 (PMC10065194; doi:10.3389/fgene.2023.1086802)
Supplement: Supplementary file 1 [file Presentation1.pdf]

文章编号:1674 －5205(2022)03-0019-(012)
DOI:10.16290/j.cnki.1674-5205.2022.03.015

论《民法典》与《个人信息保护法》的关系

程　啸

(清华大学法学院 北京 100062)

〔摘　要〕《民法典》与《个人信息保护法》是我国个人信息保护法律体系中最重要最基本的法律,它们均以保护个人信息权益,协调个人信息的保护与利用为目的。由于《民法典》与《个人信息保护法》存在调整范围与调整方法上的差异,故此,不能简单地将它们的关系界定为一般法与特别法的关系或者并存的基本法,而应当进行类型化分析。具体而言,《民法典》与《个人信息保护法》的关系可以分为四种类型:其一,《个人信息保护法》的规定对《民法典》相关规定进行了充实与丰富发展;其二,《个人信息保护法》通过转介条款或相关规定指向了《民法典》的相关规定;其三,《个人信息保护法》的规定相对于《民法典》的规定是特别规定而应当优先适用;其四,《个人信息保护法》与《民法典》有不同的规范目的而分别适用于不同的情形。

〔关键词〕民法典;个人信息保护法;个人信息权益;个人信息处理

Abstract: China Civil Code and China Personal Information Protection Law are the most important and basic laws in Chinese personal information protection legal system. Both of them aim to protect the rights and interests of personal information and give consideration to the protection and utilization of personal information. Due to the differences in the scope and methods of adjustment, the relationship between China Civil Code and China Personal Information Protection Law cannot be simply defined as the relationship between the general law and the special law or the coexisting basic law, but should be analyzed according to the different purposes and functions of the norms. Specifically, The applicable relationship between China Civil Code and China Personal Information Protection Law can be divided into four forms: Firstly, the provisions of China Personal Information Protection Law enrich and develop the relevant provisions of China Civil Code; Secondly, the provisions of China Personal Information Protection Law are transferred to the relevant provisions of China Civil Code; Thirdly, the China Personal Information Protection Law L and China Civil Code apply to different situations because they have different normative purposes; Fourth, the provisions of China Personal Information Protection Law are special provisions relative to China Civil Code and should be applied preferentially.

Key Words: Civil Code; Personal Information Protection Law; rights and interests of personal information; processing of personal information

中图分类号: D923.8　**文献标识码:** A

一、问题的提出

如果以 2005 年《刑法修正案(五)》增设"窃取、收买、非法提供信用卡信息罪"作为我国个人信息保护法律制度建立的起点,在历经十六年的发展之后,随着《个人信息保护法》的颁布,一套完备的个人信息保护法律体系已在我国建成。该体系由《民法典》《个人信息保护法》等法律,《互联网信息服务管理办法》《关键信息基础设施安全保护条例》等行政法规,最高人民法院《关于审理使用人脸识别技术处理个人信息相关民事案件适用法律若干问题的规定》《关于审理利用信息网络侵害人身权益民事纠纷案件适用法律若干问题的规定》等司法解释,《儿童

收稿日期: 2021－11－30
基金项目: 国家社科基金重大项目(18ZDA146)"大数据时代个人数据保护与数据权利体系研究";清华大学自主科研计划项目(2021THZWYY02)"个人信息权益研究"
作者简介: 程啸,清华大学法学院教授、法学博士。

(C)1994-2022 China Academic Journal Electronic Publishing House. All rights reserved.　http://www.cnki.net

个人信息网络保护规定》等部门规章及规范性文件、国家标准组成。其中，最重要也最核心的法律就是《民法典》与《个人信息保护法》。

《民法典》第111条第1句规定"自然人的个人信息受法律保护。"同时，《民法典》人格权编第六章"隐私权和个人信息保护""在现行有关法律规定的基础上，进一步强化对隐私权和个人信息的保护，并为下一步制定个人信息保护法留下空间"。[1] 该章用六个条文(第1034－1039条)分别对个人信息的概念、处理的含义、私密信息与非私密信息的法律适用、个人信息的合理使用、个人信息权益的内容、信息处理者的义务等作了规定。在《民法典》施行八个月后颁布的《个人信息保护法》是我国第一部个人信息保护方面的专门法律，该法首次提出了"个人信息权益"的概念，将保护个人信息权益作为立法目的之一，明确禁止任何组织、个人侵害自然人的个人信息权益。《个人信息保护法》第二章、第三章详细规定了公民个人信息处理规则，第四章与第五章分别规定了公民个人对个人信息处理者的权利——包括知情权、决定权、查阅复制权、个人信息可携带权、更正补充权、删除权、解释说明权等——以及个人信息处理者的义务。此外，还细致地规定了非法处理个人信息的法律责任，尤其是侵害个人信息权益的侵权责任。

在《民法典》与《个人信息保护法》都对个人信息保护作出规定的情形下，二者是何种关系，司法和执法中应当如何适用，值得研究。一种观点认为，《个人信息保护法》是一部保护个人信息的综合性立法，其所包含的个人信息的私法规则与《民法典》构成特别法与一般法的关系，《民法典》确立了个人信息的性质及其在民事权利体系中的位置，故此，《个人信息保护法》的适用需要在《民法典》的体系框架中展开。[2] 另一种观点认为，《个人信息保护法》是保护个人信息的基本立法，其调整范围、义务主体和执行机制均不同于《民法典》。故此，它不是《民法典》的特别法或下位法，《个人信息保护法》和《民法典》一样都是基本法。[3]

正确界定《民法典》与《个人信息保护法》的关系，不仅有利于构建科学合理的个人信息保护法律体系，而且对于司法与执法实践中法院与履行个人信息保护职责的部门正确适用法律处理个人信息纠纷至关重要，故此，本文将就《民法典》与《个人信息保护法》的关系问题进行研究，以供理论界与实务界参考。

二、《民法典》与《个人信息保护法》适用关系的四种类型

要正确认识《民法典》与《个人信息保护法》的关系，就有必要了解这两部法律在个人信息保护的目的、调整范围与保护方法等方面的异同。首先，必须明确的一点是，个人信息权益是自然人就其个人信息而享有的受到法律保护的利益。《个人信息保护法》更是在第1条就将"保护个人信息权益"与"促进个人信息合理利用"明确为立法目的。显然，保护作为民事权益中的人格权益的个人信息权益是《民法典》和《个人信息保护法》的共同目标，在这一点上，二者完全一致。《民法典》与《个人信息保护法》之所以要保护个人信息权益，归根结底，就是为了贯彻《宪法》尊重和保护人权，保护人格尊严、人身自由、通信自由与通信秘密的规定，落实党的十九大明确提出的要保护人民人身权、财产权、人格权的要求，[4] 更好地满足人民日益增长的美好生活需要，维护人民权益，不断增加人民群众获得感、幸福感和安全感，促进人的全面发展。[5]

虽然《民法典》与《个人信息保护法》都以保护个人信息权益，协调个人信息保护与利用关系为目的，但是它们在调整范围与调整方法上存在非常明显的差异。一方面，它们的调整范围不同。《民法典》是民事领域的基本性、综合性法律，调整的是平等主体的自然人、法人和非法人组织之间的人身关系和财产关系(第2条)。《民法

[1] 王晨《关于〈中华人民共和国民法典(草案)〉的说明》，中央人民政府网站，http://www.gov.cn/xinwen/2020－05/22/content_5513931.htm#1。2021年11月16日访问。

[2] 王利明《论〈个人信息保护法〉与〈民法典〉的适用关系》，《湖湘法学评论》2021年第1期，第25－35页。

[3] 周汉华《个人信息保护的法律定位》，《法商研究》2020年第3期，第54页。

[4] 《全国人民代表大会宪法和法律委员会关于〈中华人民共和国个人信息保护法(草案)〉审议结果的报告》，中国人大网，http://www.npc.gov.cn/npc/c30834/202108/a528d76d41c44f33980eaffe0e329ffe.shtml。2021年11月18日访问。

[5] 王晨《关于〈中华人民共和国民法典(草案)〉的说明》，中央人民政府网站，http://www.gov.cn/xinwen/2020－05/22/content_5513931.htm#1。2021年11月16日访问。

(C)1994-2022 China Academic Journal Electronic Publishing House. All rights reserved.　http://www.cnki.net

典》中的个人信息保护制度调整的也是平等主体的自然人、法人与非法人组织之间的个人信息处理关系,不包括不平等的主体之间的个人信息处理关系,如国家机关为履行法定职责而处理自然人的个人信息。[6] 《个人信息保护法》与《民法典》不同,它调整的是个人信息处理者处理自然人的个人信息的活动,只要是信息能力不平等的主体之间的个人信息处理活动,都要受到《个人信息保护法》的规范。[7] 故此,作为信息能力平等的主体之间即自然人之间因个人或者家庭事务而处理个人信息的情形,被完全排除在《个人信息保护法》的调整范围之外(第 72 条第 1 款),仍由《民法典》调整。

另一方面,《民法典》与《个人信息保护法》所采取的调整方法有异。《民法典》对个人信息保护的规定集中于人格权编。由于人格权编调整的是"因人格权的享有和保护产生的民事关系"(第 989 条),故此《民法典》主要规定个人信息权益的享有和保护,包括个人信息权益与隐私权的关系(第 1034 条第 3 款)、处理个人信息应遵循的原则和条件(第 1035 条)、处理个人信息的免责事由(第 1036 条)、个人信息权益的内容(第 1037 条)以及处理者的义务(第 1038 条)。然而,《个人信息保护法》是对个人信息处理活动进行全过程、全方位的规范,故此,该法不仅对个人信息处理的基本原则、个人信息处理规则(尤其是告知同意规则)、个人在个人信息处理活动中的权利、个人信息处理者的义务和法律责任等都有详细的规定,并且侧重于从预防的角度作出规定。这与《民法典》主要是事后补救即从侵权责任的角度作出规定,有很大的差别。

正是由于《民法典》与《个人信息保护法》对个人信息保护的规定既有共性也有差异,故此,无论将《个人信息保护法》看作《民法典》的民事特别法,还是认为它们都属于基本法的观点,都是片面的、不妥当的。正确的做法是对《民法典》与《个人信息保护法》的关系采取类型化的思考方法,具体而言,可以将它们之间的关系分为以下四种类型:第一,《个人信息保护法》对《民法典》的相关规定进行了丰富发展,作出了更具体、更详细的规定。第二,《个人信息保护法》对相关民事问题无须作出规定,而是通过转介条款或相关规范指向《民法典》。第三,《个人信息保护法》与《民法典》的某些规定虽然规范对象相同,但是由于规范目的不同,故此二者适用的情形不同,并行不悖。第四,相对于《民法典》的一般性规定而言,《个人信息保护法》对相关问题的规定属于特别规定,故此应当优先适用《个人信息保护法》的规定。下文对这四种类型的关系逐一加以论述。

三、《个人信息保护法》中丰富发展《民法典》相关规定的规范

在我国《民法典》编纂时,立法机关就已经充分认识到,不可能在《民法典》中对个人信息保护问题作出非常具体详尽的规定,只能对一些原则的、根本的问题作出规定,如明确个人信息受到法律保护、规定个人信息处理的基本原则、不区分个人信息的处理者与控制者、建立个人信息处理中的告知同意这一基本规则等。至于细节的、具体的内容则留待《个人信息保护法》作出规定。在《个人信息保护法》起草中,立法机关也始终关注如何处理好其与《民法典》等法律的衔接、细化,充实个人信息保护制度规则的问题。因此,在《民法典》颁布之后制定的《个人信息保护法》中有相当多的规定是对《民法典》规定的丰富发展,主要体现在:对个人信息处理的基本原则的丰富发展、对告知同意规则的细致性规定,对个人信息含义的界定,对个人信息处理与个人信息处理者的规定、个人信息处理的合法根据的细化规定、丰富发展个人在个人信息处理活动中的权利等。

(一)对个人信息处理基本原则的丰富发展与告知同意规则的细化

《民法典》明确规定的个人信息处理的基本原则有三项,即合法、正当与必要原则(第 1035 条)。《个人信息保护法》在《民法典》这一规定的基础上,对个人信息处理的基本原则做了全面详尽的规定。首先,对合法原则、正当原则进行了细化。《个人信息保护法》第 5 条规定处理者"不得通过误导、欺诈、胁迫等方式处理个人信息";第 10 条规定"任何组织、个人不得非法收集、使用、加工、传输他人个人信息,不得非法买卖、提供或者公开他人

〔6〕《民法典》第 1039 条关于国家机关、承担行政职能的法定机构及其工作人员不得泄露和非法提供隐私和个人信息的规定,本来不属于《民法典》的内容,只是考虑到这个问题很重要,从实用主义的角度出发才作了规定。参见黄薇主编《中华人民共和国民法典人格权编解读》,中国法制出版社 2020 年版,第 230－231 页。

〔7〕丁晓东《个人信息保护原理与实践》,法律出版社 2021 年版,第 191 页。

(C)1994-2022 China Academic Journal Electronic Publishing House. All rights reserved.　　http://www.cnki.net

个人信息;不得从事危害国家安全、公共利益的个人信息处理活动。”其次,新增公开透明原则、诚信原则、目的限制原则、质量原则以及责任原则作为个人信息处理的基本原则(第5条至第9条)。再次,《个人信息保护法》第6条第2款将《民法典》第1035条中的“不得过度处理”细化为“收集个人信息,应当限于实现处理目的的最小范围,不得过度收集个人信息”。

告知同意规则是个人信息处理中最基本的规则,依据这一规则,除非法律、行政法规另有规定,否则个人信息处理者在处理个人信息前必须向个人信息被处理的个人告知相应的事项并取得个人的同意,否则该处理活动就是非法的。《民法典》第1035条规定的“征得该自然人或者其监护人同意”以及“公开处理信息的规则”“明示处理信息的目的、方式和范围”中已经蕴含了告知同意规则,但规定得非常简单。《个人信息保护法》在第二章与第三章对告知同意规则予以详细规定,尤其是对告知的方式、告知的事项、免于告知的情形、个人同意的有效要件、同意的方式、同意的撤回等,都作了细致完备的规定。

(二)对个人信息含义的丰富发展

《民法典》第1034条第2款规定“个人信息是以电子或者其他方式记录的能够单独或者与其他信息结合识别特定自然人的各种信息,包括自然人的姓名、出生日期、身份证件号码、生物识别信息、住址、电话号码、电子邮箱、健康信息、行踪信息等。”显然,《民法典》对个人信息采取了“识别说”。《个人信息保护法》并未逐一列举个人信息的类型,但对个人信息的界定采取了“关联说”。该法第4条第1款规定“个人信息是以电子或者其他方式记录的与已识别或者可识别的自然人有关的各种信息,不包括匿名化处理后的信息。”就《民法典》与《个人信息保护法》对个人信息界定的不同,有观点认为,《个人信息保护法》对个人信息的界定不仅保留了识别性标准,还增加了关联性标准,将信息内容本身可能不具有识别性,但已与特定自然人形成稳定关联的信息同样纳入个人信息的范畴,对个人信息的界定明显更加宽泛。[8] 笔者认为,《个人信息保护法》对个人信息的界定是对《民法典》规定的发展和完善,二者实质是相同的,不存在矛盾冲突。换言之,无论是采取识别说还是关联说,所界定的个人信息的范围都是相同的。从识别说的角度来界定个人信息,只有可以直接或间接识别的特定自然人的信息才是个人信息。这是从信息本身出发,看能否从中找到与特定自然人的关联性。以关联说来界定个人信息则是从信息主体出发,认为只有与已识别或可识别的自然人相关的信息才是个人信息。那些与已识别或可识别的自然人无关的信息,本身也无法直接或间接识别特定的自然人,不属于个人信息。例如,个人信息处理者A公司已经知道了特定的自然人张三,此时,对A公司而言,关于张三的所有信息都是个人信息,A公司处理张三的信息时必须遵循《个人信息保护法》的要求。例如,张三是A公司的员工,其工作证号码是:A1998990126。由于该号码就是A公司分配给张三的,故此对于A公司而言,该工作证号码就是张三的个人信息。可是,对于其他人来说,并不知道该工作证号码是张三的。依据关联说,A1998990126就不是张三的个人信息。同时,从识别说来看,仅仅这样一组号码,对于A公司之外的其他处理者而言是无法直接识别出张三的,除非将该号码与其他信息结合。由此可见,个人信息定义中的关联说更强调的是个人信息的相对性,即有些信息是否属于个人信息是相对而言的,即对于某些处理者来说是个人信息,对另一些处理者就不是个人信息。个人信息定义中的识别说更强调的是从信息本身出发,而是否可以直接或者间接识别特定自然人同样是具有相对性的。

(三)个人信息处理类型的丰富与个人信息处理者的界定

《民法典》第1035条第2款对个人信息处理作了规定,即“包括个人信息的收集、存储、使用、加工、传输、提供、公开等”。同时,《民法典》没有采取区分个人信息处理者与个人信息控制者的立法模式,而统一采取了“个人信息处理者”的概念(第1030条、第1037条以及第1038条)。《个人信息保护法》在《民法典》的基础上,对个人信息处理和个人信息处理者作了更细致的规定。首先,《个人信息保护法》第4条第2款在《民法典》第1035条第2款的基础上新增了一类典型的处理情形即“删除”。其次,个人信息处理者在个人信息保护中至关重要,他

〔8〕张新宝:《〈中华人民共和国个人信息保护法〉释义》,人民出版社2021年版,第42页;孙莹主编:《个人信息保护法条文解读与适用要点》,法律出版社2021年版,第13页。

(C)1994-2022 China Academic Journal Electronic Publishing House. All rights reserved.　http://www.cnki.net

既是个人行使个人信息权益所指向的相对人，也是依法负有各种个人信息保护义务的主体，故此，界定个人信息处理者至关重要。《个人信息保护法》第 73 条第 1 项对个人信息处理者作了明确界定，即“个人信息处理者，是指在个人信息处理活动中自主决定处理目的、处理方式的组织、个人”。这是因为，处理目的说明了个人信息处理为何发生，即为什么处理者要处理个人信息; 处理方式则说明了个人信息处理如何发生，即处理者将如何处理个人信息。能够自主决定这两点的组织或个人就是个人信息处理者。再次，《个人信息保护法》区分个人信息的处理者与受委托处理个人信息的受托人。在接受委托处理个人信息时，受托人并不是自主决定个人信息处理目的和处理方式的人，所以其不是个人信息处理者，不可能如个人信息处理者那样负有各种义务和责任。为了正确区分二者，《个人信息保护法》第 59 条规定，接受委托处理个人信息的受托人，应当依照本法和有关法律、行政法规的规定，采取必要措施保障所处理的个人信息的安全，并协助个人信息处理者履行本法规定的义务。

(四) 对个人信息处理的合法根据的具体规定

《民法典》从两个方面对个人信息处理活动合法与否作了回答。一方面，第 1035 条第 1 款规定了合法的个人信息处理应具备的条件，即处理个人信息的，应当遵循合法、正当、必要原则，不得过度处理，并应征得该自然人或者其监护人同意。另一方面，第 999 条和第 1036 条又分别从个人信息的合理使用与免责事由的角度对合法的个人信息处理作了规定。依据《民法典》第 999 条，为公共利益实施新闻报道、舆论监督等行为的，可以合理使用民事主体的个人信息，使用不合理侵害民事主体人格权的，应当依法承担民事责任; 而第 1036 条又规定了三种情形中处理个人信息的，行为人不承担民事责任，即在该自然人或者其监护人同意的范围内合理实施的行为、合理处理合法公开的个人信息等。

《个人信息保护法》在《民法典》上述规定的基础上，从处理规则的角度对个人信息处理的合法根据作了系统全面的规定。该法第 13 条第 1 款详细列举了所有的合法处理个人信息的情形，具体来说，分为两类: 一是取得个人同意处理个人信息; 另一类是法律或行政法规规定不需要取得个人同意就可以处理个人信息的情形，具体包括六种，其中新增的合法性根据如为订立、履行个人作为一方当事人的合同所必需，或者按照依法制定的劳动规章制度和依法签订的集体合同实施人力资源管理所必需; 为履行法定职责或者法定义务所必需; 为应对突发公共卫生事件，或者紧急情况下为保护自然人的生命健康和财产安全所必须等情形。

(五) 丰富发展了个人在个人信息处理活动中的权利

《民法典》规定了个人针对个人信息处理者的四种权利，即知情同意权(第 1035 条、第 1038 条) 、查阅复制权(第 1037 条第 1 款第 1 句) 、更正权(第 1037 条第 1 款第 2 句) 、删除权(第 1037 条第 2 款) 。《个人信息保护法》在《民法典》的基础上，专章详尽规定了个人在个人信息处理活动中的权利，不仅明确了这些权利的性质，而且丰富了权利的类型以及救济程序。(1) 《个人信息保护法》第四章采取了“个人在个人信息处理活动中的权利”的称谓，明确了这些权利在性质上属于请求权即手段性权利或救济性权利，是作为信息主体的个人在个人信息处理活动中针对个人信息处理者享有的权利。(2) 《个人信息保护法》为这些权利建立了实现和保障机制，即第 50 条要求个人信息处理者建立便捷的个人行使权利的申请受理和处理机制。拒绝个人行使权利的请求的，应当说明理由。如果个人信息处理者拒绝个人行使权利的请求，个人可以依法向人民法院提起诉讼。(3) 新增了四种个人针对个人信息处理者的权利，即个人对其个人信息处理的知情权与决定权(第 44 条) 、个人信息的可携带权(第 45 条第 3 款) 、个人信息的补充权(第 46 条) 以及个人信息处理规则的解释说明权(第 48 条) 。(4) 对权利行使的具体情形作了规定，包括: 一方面，明确规定了个人不得向个人信息处理者行使查阅复制权的情形(第 45 条第 1 款) ; 另一方面，规定了个人行使个人信息删除权的五种具体情形并规定了在这些情形中个人信息处理者负有主动删除的义务，还规定了不能删除时个人信息处理者应当停止除存储和采取必要的安全保护措施之外的处理。(5) 规定了自然人死亡的，其近亲属为了自身合法、正当利益，可以对死者的相关个人信息行使本章规定的查阅、复制、更正、删除等权利; 但死者生前另有安排的除外。

四、《个人信息保护法》通过转介条款指向《民法典》的相应规定

《个人信息保护法》是个人信息保护领域的综合性法律，其综合运用民法、行政法、国际法、刑法等多种部门

(C)1994-2022 China Academic Journal Electronic Publishing House. All rights reserved.　http://www.cnki.net

法的方法对个人信息处理活动进行全方位的调整，从而保护个人信息权益，促进个人信息的合理利用。在我国已经颁布了系统性规范各类民事主体的人身关系和财产关系的《民法典》的情形下，《个人信息保护法》在运用民法的调整方法时，显然无需重复《民法典》的规定，而只需要通过相应的转介条款或规范指向《民法典》即可，由《民法典》的规定来实现对这些问题的具体调整。这些情形中比较典型的如对个人信息处理活动中的格式条款的规范、委托处理个人信息中对委托合同的规范以及共同处理个人信息的连带责任等。

(一) 个人信息处理规则与格式条款的规制

个人信息处理规则，也称"隐私政策"(privacy policy)，它是指个人信息处理者自行制定并公开的关于如何处理个人信息的规则，主要包括个人信息处理者的名称或者姓名和联系方式；个人信息的处理目的、处理方式，处理的个人信息种类、保存期限；个人行使法律规定的针对个人信息处理者的权利的方式和程序等。隐私政策最初起源于美国，当时主要是因为个人信息保护立法不完善，企业便自行对那些尚未立法的领域通过制定隐私政策的方式来进行自律化管理，从而满足个人信息处理公开透明原则的要求。〔9〕 在我国，《民法典》第 1035 条第 1 款以及《个人信息保护法》第 7 条、第 17 条第 3 款明确规定了个人信息处理者必须公开个人信息处理规则。《个人信息保护法》第 31 条第 2 款还规定，个人信息处理者处理不满十四周岁的未成年人个人信息的，应当制定专门的个人信息处理规则。

个人信息处理规则是个人信息处理者单方面制定的，并未与个人协商，里面包含的不限于单纯的向个人履行告知义务的内容，还可能包括个人信息处理者对于其与个人之间的权利义务的规定，如施加给个人的义务，免除或减轻自己责任的规定、限制或者排除个人权利的内容等。此时，为了保护个人信息权益，就必须适用《民法典》关于格式条款的规定来评价个人信息处理规则中处理者单方面就其与个人之间的权利义务责任关系作出的规定。

一方面，对于个人信息处理者要求个人表示同意的个人信息处理规则，因个人的同意而使得该处理规则纳入个人与处理者之间的网络服务合同当中的，必须适用《民法典》第 496 条第 2 款的规定，即处理者应当遵循公平原则确定当事人之间的权利和义务，并采取合理的方式提示个人注意免除或者减轻其责任等与个人有重大利害关系的条款，按照个人的要求，对该条款予以说明。如果个人信息处理者不履行提示或者说明义务，致使个人没有注意或者理解与其有重大利害关系的条款的，个人可以主张该条款不能成为网络服务合同的内容。另一方面，对于不需要取得个人同意的个人信息处理规则，无论个人信息处理者是企业还是国家机关抑或其他民事主体，如果个人信息处理规则中存在《民法典》第一编第六章第 3 节和第 506 条规定的无效情形，或者处理者通过个人信息处理规则不合理地免除或者减轻其责任、加重个人的责任、限制个人的主要权利，或者处理者排除个人的主要权利的，那么这些个人信息处理规则就是无效的。

(二) 个人信息共同处理者的连带责任

个人信息的共同处理，是指两个以上的个人信息处理者共同决定个人信息的处理目的和处理方式(《个人信息保护法》第 20 条第 1 款第 1 句)。共同处理个人信息时，各个处理者之间应当约定各自的权利义务，但这种约定并不影响个人向其中任何一个处理者要求行使《个人信息保护法》规定的权利(《个人信息保护法》第 20 条第 1 款第 2 句)。如果数个处理者在共同处理个人信息时，侵害了公民个人信息权益并造成损害，应当如何承担责任呢？对此，《个人信息保护法》第 20 条第 2 款规定，共同处理者"应当依法承担连带责任"。该规定就是所谓的转引条款，所谓"依法"当然是指依据《民法典》关于连带责任的规定。《民法典》第 1168 条至第 1171 条对多数人侵权的连带责任作了规定，包括共同加害行为(第 1168 条)，教唆帮助行为(第 1169 条)，共同危险行为(第 1170 条)以及无意思联络的数人侵权(第 1171 条)。就构成共同加害行为的共同处理个人信息的侵权行为而言，最常见的就是非法买卖个人信息，即信息处理者以买卖或者其他方式非法向第三人提供个人信息，进而给自然人造成

〔9〕 王叶刚《网络隐私政策法律调整与个人信息保护：美国实践及其启示》，《环球法律评论》2020 年第 2 期，第 150－152 页。

(C)1994-2022 China Academic Journal Electronic Publishing House. All rights reserved. http://www.cnki.net

损害。此时,无论是出卖人还是买受人都属于共同故意实施侵权行为,应当承担连带责任。如果个人信息处理者的共同处理行为构成教唆帮助侵权行为,教唆人、帮助人应当与行为人承担连带责任。上述两类分别构成共同加害行为、教唆帮助行为的情形,虽然也可以被纳入共同处理个人信息的情形,但仅仅这两种情形并未涵盖共同处理个人信息的全部类型。因为在上述两类情形中,数个处理者一开始就是以追求侵害自然人个人信息权益为目的而共同实施非法处理个人信息的违法行为。然而,因共同处理个人信息侵害个人信息权益的情形,可能并非都是如此,有可能的情形是:多个处理者虽然共同决定了个人信息的处理目的和处理方式,都依法取得了个人同意,且处理目的和处理方式也都不存在违法的情形,即共同处理者并非一开始就追求侵害个人信息权益的非法目的。但是,他们在共同处理活动的过程中都超越了告知个人的处理目的与处理方式,增加了新的处理目的和方式,或者在处理中都没有尽到个人信息安全保护义务以致个人信息被他人窃取或篡改,从而侵害个人信息权益并造成损害。例如,作为共同处理者的 A 公司与 B 公司都违反了《民法典》第 1038 条第 2 款,未采取技术措施和其他必要措施确保其收集、存储的个人信息安全,以致个人信息被泄露,但现在不清楚究竟是哪一个处理者的行为实际给个人造成了损害。此时,无论是否能够查明共同处理者中的哪一个实际泄露了个人信息,都可以适用《民法典》第 1170 条规定的共同危险行为,[10]共同处理者要向遭受损害的自然人承担连带赔偿责任。如果某个处理者能够证明并非其处理行为实际造成了损害,则可以免责。再如,共同处理者均违反了法定义务或约定义务,以致发生个人信息泄露,且每个行为都足以造成信息主体的同一损害,或者某些行为足以造成信息主体的全部的同一损害,某些只能造成信息主体的部分的同一损害。如果是前一种情形即所有的处理者的行为均足以造成信息主体的同一损害,那么可以依据《民法典》第 1171 条的规定,令这些处理者承担连带责任。但如果是后一种情形,依据《民法典》第 1172 条,这些处理者应承担按份责任,即能够确定责任大小的,各自承担相应责任;难以确定责任大小的,平均承担责任。

(三) 个人信息委托处理中的委托合同与相应的民事责任

个人信息的委托处理是个人信息处理活动中常见的情形,它是指个人信息处理者将处理个人信息的事务委托给其他的组织或个人,双方成立委托合同关系,一方是委托人,另一方是受托人,由受托人为委托人处理个人信息。委托人可以特别委托受托人对某一种类的个人信息实施某种处理活动(如仅仅是存储或加工),也可以委托受托人对某些种类的个人信息实施多种处理活动(如既存储,也加工、分析等)。《个人信息保护法》第 21 条对个人信息的委托处理作了规定,包括对于委托人和受托人约定的内容、受托人的义务、委托合同不生效、无效、被撤销或终止后的受托人返还或删除个人信息的义务以及不得转委托等。显然,除了《个人信息保护法》第 21 条的规定,对于个人信息委托处理的民法规范必须适用《民法典》。(1) 委托人与受托人的权利义务适用委托合同的规定。《个人信息保护法》第 21 条第 1 款对委托人与受托人应当约定的内容作了规定,即双方应当约定"委托处理的目的、期限、处理方式、个人信息的种类、保护措施以及双方的权利和义务等"。从这一规定可知,立法者将"委托处理的目的、期限、处理方式、个人信息的种类、保护措施"与"双方的权利义务"分开表述。这是因为:前者涉及个人信息权益的保护,这些约定既是委托方与受托方对"委托事务"的具体约定,也是法律规定的义务,故此,它们属于个人信息处理者委托他人处理个人信息的委托合同必须具备的条款。至于"双方的权利义务"则是指委托合同中委托人和受托人的主要权利义务,具体而言在当事人有约定的时候适用约定,没有约定或约定不明的,应当适用《民法典》合同编第二十三章关于委托合同中委托人和受托人主要权利义务的规定。(2) 紧急情况下的个人信息处理的转委托适用《民法典》的规定。委托合同是基于委托人对受托人的信赖而委托受托人处理事项所产生的民事法律关系。受托人负有亲自处理委托事务的义务,而不得任意转委托他人。《民法典》第 923 条规定"受托人应当亲自处理委托事务。经委托人同意,受托人可以转委托。转委托经同意或者追认的,委托人可以就委托事务直接指示转委托的第三人,受托人仅就第三人的选任及其对第三人的指示承担责任。转委托

〔10〕 阮神裕《民法典视角下个人信息的侵权法保护——以事实不确定性及其解决为中心》,《法学家》2020 年第 4 期,第 34 页。

(C)1994-2022 China Academic Journal Electronic Publishing House. All rights reserved.　http://www.cnki.net

未经同意或者追认的,受托人应当对转委托的第三人的行为承担责任;但是,在紧急情况下受托人为了维护委托人的利益需要转委托第三人的除外。"同理,在个人信息的委托处理中,受托人也不能随意转委托。《个人信息保护法》第 21 条第 3 款规定,未经个人信息处理者同意,受托方不得转委托他人处理个人信息。有疑问的是,如果存在紧急情况,受托人能否转委托? 此时可以适用《民法典》第 923 条的规定,即在紧急情况下受托人为了维护作为委托人的个人信息处理者的利益,可以转委托给第三人。

再次,《个人信息保护法》没有对委托处理个人信息时,受托人侵害自然人的个人信息权益的情形下,委托人与受托人之间如何承担责任作出规定。因此,需要适用的是《民法典》的规定。具体而言,一方面,委托处理个人信息时,受托人因处理行为侵害个人信息权益造成损害的,属于定作人责任,应适用《民法典》第 1193 条,即只有当委托人就个人信息处理存在定作、指示、选任的过错的,才需要承担相应的责任。另一方面,在委托他人处理个人信息时,由于委托人和受托人具有委托合同关系,双方会对权利义务作出约定,故此,受托人在向受害人赔偿后,可以依据约定向委托人进行追偿。此外,如果因为受托人违反合同而处理个人信息且因此给委托人造成损失的,委托人就该损失可以向受托人要求赔偿。此时,适用《民法典》第 929 条,即"有偿的委托合同,因受托人的过错造成委托人损失的,委托人可以请求赔偿损失。无偿的委托合同,因受托人的故意或者重大过失造成委托人损失的,委托人可以请求赔偿损失。受托人超越权限造成委托人损失的,应当赔偿损失。"

五、《民法典》与《个人信息保护法》中因规范目的不同而分别适用的规定

如前所述,《民法典》与《个人信息保护法》对个人信息保护的调整方法存在差异,《民法典》侧重的是对个人信息权益被侵害的民事责任的规定,即侵害行为发生后的保护与救济,而《个人信息保护法》由于对个人信息处理活动进行全方位的规范,尤其是可以有大量的公法性规范来确立个人信息处理者的义务,从而通过对个人信息处理活动的各种严格规范以及施加给个人信息处理者相应的义务来防止出现非法处理个人信息以及侵害个人信息权益的行为。所以,《个人信息保护法》在个人信息权益保护上更侧重防患于未然,而非亡羊补牢。这就使得《民法典》与《个人信息保护法》中的有些规范虽然都是解决相同的问题,但是由于规范目的不同,因此适用情形也不同,并行不悖。这种关系类型主要包括:《民法典》中的私密信息与《个人信息保护法》中的敏感信息、对合法公开的个人信息处理的规范以及关于死者的个人信息的规范。

(一) 私密信息与敏感的个人信息

私密信息与非私密信息、敏感的个人信息与非敏感的个人信息是对个人信息的两种重要分类,分别是《民法典》与《个人信息保护法》中的分类方法。一些人曾认为,《个人信息保护法》除规定敏感的个人信息外,还应当继续采取《民法典》中私密信息的分类。(1) 这种观点的错误之处在于,没有认识到《民法典》与《个人信息保护法》中这两种个人信息分类的规范目的差异。私密和非私密的个人信息是《民法典》从民事权益保护和法律适用的角度对个人信息进行的分类。因为私密信息属于隐私,所以既可以适用隐私权保护的规定,也可以适用个人信息保护的规定。故此,《民法典》第 1034 条第 3 款规定:"个人信息中的私密信息,适用有关隐私权的规定;没有规定的,适用有关个人信息保护的规定。"此外,私密信息与非私密信息的区分仅仅在平等民事主体的侵权纠纷中有意义。例如,对于私密信息的侵害,由于可以适用隐私权保护的规定,所以被侵权人可以行使人格权保护请求权,向法院申请人格权禁令,但是对于非私密信息则不能适用人格权禁令程序。[11] 此外,哪些个人信息是私密信息,也必须从社会公众的一般认知和价值权衡的角度出发,根据案件具体事实逐一认定。(2) 敏感的个人信息与非敏感的个人信息是《个人信息保护法》从个人信息处理者的义务角度作出的区分,由于敏感的个人信息与自然人的人格尊严、人格自由等基本权利和重大人身财产权益具有极为密切的联系,故此,《个人信息保护法》对个人信息处理者处理敏感的个人信息提出了极严格的要求,包括:处理者只有具有特定的目的和充分的必要性并采取严格的保护措施,才能处理敏感的个人信息(第 28 条第 2 款);处理敏感的个人信息必须取得个人的单独同意

〔11〕 程啸:《论我国民法典中的人格权禁令制度》,《比较法研究》2021 年第 3 期,第 145 页。

(C)1994-2022 China Academic Journal Electronic Publishing House. All rights reserved.　http://www.cnki.net

(第 29 条);处理者在处理前必须进行个人信息保护影响评估并对处理情况进行记录等(第 55 条第 1 项)。正是由于敏感信息和非敏感信息是为了确定不同的个人信息处理规则而对个人信息作出的区分,故此,该分类仅适用于个人信息保护法所调整的个人信息处理行为,而不适用于自然人因个人或者家庭事务而处理个人信息的活动。区分并明确列举敏感的个人信息,对于自然人、个人信息处理者以及履行个人信息保护职责的部门来说,都非常有必要。对自然人而言,这使其可以更充分意识到敏感个人信息的重要性,继而采取有效的自我保护行动;对个人信息处理者而言,在明确了哪些个人信息是敏感个人信息后,就能够降低履行个人信息保护义务的合规成本;对于履行个人信息保护职责的部门来说,可以集中执法资源对侵害敏感个人信息的违法行为进行查处,提高执法效率。

(二)合法公开的个人信息的处理

所谓合法公开的个人信息就是指个人自行公开的或者其他已经合法公开的个人信息。《民法典》第 1036 条第 2 项与《个人信息保护法》第 13 条、第 27 条都对合法公开的个人信息作了规定。但是,它们的规范目的却存在差别。因为《民法典》是从处理个人信息民事责任免责事由的角度加以规定的,只要处理者是合理处理已经合法公开的个人信息,就可以不承担因处理个人信息而产生的民事责任。故此,《民法典》第 1036 条第 2 项并未明确处理者在处理合法公开的个人信息时是否需要取得个人同意。

《个人信息保护法》是从个人信息处理规则的角度加以规定的。一方面,《个人信息保护法》第 13 条第 1 款第 6 项将《民法典》第 1036 条第 2 项的"合理处理"细化为"依照本法规定在合理的范围内处理";同时,第 13 条第 2 款还明确规定,对合法公开的个人信息的合理处理无须取得个人的同意。另一方面,《个人信息保护法》第 27 条第 3 句规定"个人信息处理者处理已公开的个人信息,对个人权益有重大影响的,应当依照本法规定取得个人同意。"该句中"对个人权益有重大影响"的表述不同于《民法典》第 1036 条第 2 项规定的"侵害其重大利益"。之所以如此,是因为,《民法典》第 1036 条第 2 项是从处理个人信息民事责任免责事由或个人信息合理使用的角度作出的规定,也就是说,只有在行为人处理合法公开的个人信息已经侵害了自然人重大利益的情形下,才能基于比例原则的考虑,优先保护重大利益,认定处理者不得以此免责。处理者应当向个人承担停止侵害、排除妨碍以及赔偿损害等侵权责任。个人负有证明处理者已经侵害其重大利益的举证责任。但是,《个人信息保护法》第 27 条第 3 句规范的对象是个人信息处理者,属于预防性或事先的规范,即处理者在处理合法公开的个人信息之前就必须对该处理活动是否对个人权益有重大影响通过进行个人信息保护影响评估而得出相应的判断(《个人信息保护法》第 55 条第 5 项),据此决定是否需要取得个人的同意。这是对个人信息处理者的要求,所以个人信息处理者要负担举证责任。

(三)死者的个人信息

《民法典》第 984 条规定"死者的姓名、肖像、名誉、荣誉、隐私、遗体等受到侵害的,其配偶、子女、父母有权依法请求行为人承担民事责任;死者没有配偶、子女且父母已经死亡的,其他近亲属有权依法请求行为人承担民事责任。"该规定中没有列出死者的个人信息。当然,死者的个人信息中属于姓名、肖像和隐私的部分,可以适用上述规定而得到保护。此外,由于私密信息属于隐私,受到隐私权的保护(《民法典》第 1034 条第 3 款),死者的私密信息保护也可以适用《民法典》第 984 条。问题是,死者的个人信息中那些并非死者的姓名、肖像或隐私的个人信息,是否受到侵权法的保护呢?笔者认为,保护这些个人信息也可以适用《民法典》第 994 条的规定。因为,《民法典》第 990 条第 2 款规定"除前款规定的人格权外,自然人享有基于人身自由、人格尊严产生的其他人格权益。"当死者的个人信息被侵害进而损害死者近亲属的人身财产权益时,死者的近亲属可以依据《民法典》第 990 条第 2 款规定的一般人格权请求保护。同时,由于《民法典》第 994 条在列举死者的人格要素时并未穷尽,而使用了"等"字兜底,故此,可将死者的个人信息中并非姓名、肖像、隐私的个人信息包括进去,即在这些个人信息遭受侵害时,死者的配偶、子女、父母等近亲属,也可以依据该条请求行为人承担民事责任。鉴此,《最高人民法院关于审理使用人脸识别技术处理个人信息相关民事案件适用法律若干问题的规定》第 15 条规定"自然人死亡后,信息处理者违反法律、行政法规的规定或者双方的约定处理人脸信息,死者的近亲属依据民法典第九百九

(C)1994-2022 China Academic Journal Electronic Publishing House. All rights reserved.　http://www.cnki.net

十四条请求信息处理者承担民事责任的,适用本规定。”

《个人信息保护法》第 49 条规定“自然人死亡的,其近亲属为了自身的合法、正当利益,可以对死者的相关个人信息行使本章规定的查阅、复制、更正、删除等权利;死者生前另有安排的除外。”既然《民法典》第 994 条已经为死者的个人信息提供了侵权法保护,为何《个人信息保护法》第 49 条还作出上述规定呢? 这是因为,《民法典》与《个人信息保护法》的规范目的不同。依据《民法典》第 994 条,只有在行为人实施了侵害死者个人信息的侵权行为后,死者的配偶、子女、父母等近亲属才有权依法请求行为人承担民事责任。《民法典》提供的是一种消极、被动的保护或者说民事责任意义上的保护。但是,《个人信息保护法》赋予了死者近亲属针对死者的个人信息可以积极行使相应权利,是一种积极、主动的保护。死者的近亲属无须在死者的隐私被侵害的情形下才能提起侵权诉讼,而是在为了自身的合法、正当利益时,直接针对死者的相关个人信息行使查阅、复制、删除等权利。〔12〕

正是由于《民法典》第 994 条与《个人信息保护法》第 49 条的规范目的不同,故此它们存在以下差异:首先,近亲属提起诉讼的顺应有无不同。依据《民法典》第 994 条,死者的姓名、肖像、名誉、荣誉、隐私、遗体等受到侵害的,其配偶、子女、父母是第一顺位的请求权人,只有当死者没有配偶、子女且父母已经死亡的,其他近亲属才有权依法请求行为人承担民事责任。《民法典》如此规定主要是考虑到保护死者的姓名、名誉等本质上是保护活着的人即近亲属的利益,而在近亲属中配偶、子女、父母与死者的关系最近,受到的不利影响最大。既然如此,在死者的配偶、子女、父母不起诉时,其他近亲属没有权利起诉。〔13〕 然而,由于死者的近亲属只有在“为了自身的合法、正当利益”时,才能针对死者的相关个人信息行使查阅、复制、更正、删除等权利,故此,《个人信息保护法》第 49 条就无须规定请求权人的顺位。其次,只要是死者的个人信息遭受了侵害,死者的近亲属就有权要求行为人承担民事责任,包括停止侵害、排除妨害、消除危险、赔偿损失、赔礼道歉等。而依据《个人信息保护法》第 49 条,死者的近亲属只是可以针对死者的相关个人信息行使《个人信息保护法》第四章规定的查阅、复制、更正、删除等权利。如果个人信息处理者拒绝个人行使权利的请求,个人可以依法向人民法院起诉。再次,死者可以通过遗嘱等排除其死后近亲属针对其个人信息行使相应的权利。但是,对于侵害死者的个人信息的侵权责任而言,不存在死者可以预先加以排除的问题。

六、《个人信息保护法》中属于《民法典》的特别规定的规范

《个人信息保护法》中有大量的民事法律规范,〔14〕这些民事法律规范与《民法典》中保护个人信息的规范之间的关系除了前述的三种情形外,还有一种就是特别法与一般法的关系。申言之,立法机关基于特殊的考虑在《个人信息保护法》中对相关问题作了特别规定,这些规定相对于《民法典》的规定就是特别法。《立法法》第 92 条规定“同一机关制定的法律、行政法规、地方性法规、自治条例和单行条例、规章,特别规定与一般规定不一致的,适用特别规定;新的规定与旧的规定不一致的,适用新的规定。”《民法典》第 11 条也规定“其他法律对民事关系有特别规定的,依照其规定。”故此,当《个人信息保护法》的规定相对于《民法典》而言是特别规定时,应当优先适用《个人信息保护法》的规定。我国《个人信息保护法》中这种特别规定主要有两项:其一,《个人信息保护法》第 69 条第 1 款关于侵害个人信息权益的侵权责任归责原则的规定;其二,《个人信息保护法》第 69 条第 2 款关于侵害个人信息权益的损害赔偿计算方法的规定。

(一) 侵害个人信息权益赔偿责任的归责原则

我国《民法典》以过错责任为侵权赔偿责任的最基本归责原则(第 1165 条第 1 款),并将过错推定责任与无过错责任限制在法律有规定的情形(第 1165 条第 2 款、第 1166 条)。同时,《民法典》第 11 条也规定“其他法律

〔12〕 对于死者近亲属行使针对死者相关个人信息的权利的具体要件的论述,参见程啸《论死者个人信息的保护》,《法学评论》2021 年第 5 期,第 22 – 23 页。

〔13〕 程啸《侵权责任法》(第 3 版),法律出版社 2021 年版,第 185 页。

〔14〕 王利明教授认为,《个人信息保护法》的民法规范约有 53 条。参见王利明《论〈个人信息保护法〉与〈民法典〉的适用关系》,《湖湘法学评论》2021 年第 1 期,第 25 页。

(C)1994-2022 China Academic Journal Electronic Publishing House. All rights reserved. http://www.cnki.net

对民事关系有特别规定的,依照其规定。”这就表明,《民法典》以及其他法律可以对某些侵权赔偿责任规定过错推定责任或无过错责任。就侵害个人信息权益的侵权赔偿责任而言,《民法典》并未规定过错推定责任或者无过错责任,故此,在《个人信息保护法》颁布之前,侵害个人信息权益的侵权赔偿责任应当适用《民法典》第 1165 条第 1 款的过错责任原则。在我国《个人信息保护法》起草过程中,就侵害个人信息权益的侵权赔偿责任究竟采取何种归责原则,存在很大争议。最终,《个人信息保护法》第 69 条第 1 款明确采取了过错推定责任,即“处理个人信息侵害个人信息权益造成损害,个人信息处理者不能证明自己没有过错的,应当承担损害赔偿等侵权责任”。立法机关之所以对侵害个人信息权益的侵权赔偿责任采取过错推定责任,主要是考虑到: 如果适用过错责任原则,就意味着被侵权人即个人需要证明侵权人即个人信息处理者存在过错,而这非常困难。因为个人信息处理活动具有很强的专业性和技术性,个人与个人信息处理者存在信息、技术、资金等能力上的不对等,无法了解个人信息处理者在处理活动中具有什么过错,更无法提出证据加以证明; 如果采取无过错责任,则过于严厉。毕竟个人信息处理活动是现代社会生活须臾不可或缺的活动,个人信息处理者的类型多种多样,处理个人信息的场景千差万别,不应当给处理者增加过多的负担。因此,只有过错推定责任最为合适,既有利于保护个人信息权益,减轻受害人的举证责任,对个人信息处理者也不过苛,其仍有机会通过证明自己没有过错而免除责任。[15] 在立法者已经基于上述考虑而作出特别规定的情形下,显然《个人信息保护法》第 69 条第 1 款相对于《民法典》第 1165 条第 1 款的过错责任原则就是特别法,应当优先适用。

(二) 侵害个人信息权益的损害赔偿确定

《民法典》第 1182 条规定 “侵害他人人身权益造成财产损失的,按照被侵权人因此受到的损失或者侵权人因此获得的利益赔偿; 被侵权人因此受到的损失以及侵权人因此获得的利益难以确定,被侵权人和侵权人就赔偿数额协商不一致,向人民法院提起诉讼的,由人民法院根据实际情况确定赔偿数额。”此外,《最高人民法院关于审理利用信息网络侵害人身权益民事纠纷案件适用法律若干问题的规定》第 12 条规定 “被侵权人为制止侵权行为所支付的合理开支,可以认定为民法典第一千一百八十二条规定的财产损失。合理开支包括被侵权人或者委托代理人对侵权行为进行调查、取证的合理费用。人民法院根据当事人的请求和具体案情,可以将符合国家有关部门规定的律师费用计算在赔偿范围内。被侵权人因人身权益受侵害造成的财产损失以及侵权人因此获得的利益难以确定的,人民法院可以根据具体案情在 50 万元以下的范围内确定赔偿数额。”在《个人信息保护法》颁布之前的司法实践中,法院依据上述规定处理了不少侵害个人信息权益的案件。[16]

《个人信息保护法》第 69 条第 2 款规定 “前款规定的损害赔偿责任按照个人因此受到的损失或者个人信息处理者因此获得的利益确定; 个人因此受到的损失和个人信息处理者因此获得的利益难以确定的,根据实际情况确定赔偿数额。”特别需要注意的是,该款使用的是“损失”一词,而没有采用《民法典》第 1182 条“财产损失”的表述。这是立法机关有意为之。所谓损失,既包括财产损失或财产损害,也包括精神损失或精神损害。[17] 也就是说,依据《个人信息保护法》第 69 条第 2 款,只要侵害个人信息权益造成损害的,无论是财产损失还是精神损失,都可以按照个人因此受到的损失或者个人信息处理者因此获得的利益确定,如果个人因此受到的损失和个人信息处理者因此获得的利益难以确定的,根据实际情况确定赔偿数额。这一规定明显不同于《民法典》第 1182 条,属于特别规定。之所以如此,原因在于: 首先,个人信息处理者的处理活动侵害个人信息权益的,如果只是侵害单纯的个人信息,而未同时构成对隐私权、名誉权、财产权等具体权利的侵害时,受害人往往没有财产损失,因为单个的个人信息的价值并不高。故此,《民法典》第 1182 条的规定才有必要。其次,侵害个人信息权益的精神损害通常只是某种心理上的焦虑或不安,或者生活上的些许不便,如骚扰电话或垃圾邮件。这种精神损害的程度

〔15〕 程啸《侵害个人信息权益的侵权责任》,《中国法律评论》2021 年第 5 期,第 61 - 63 页。

〔16〕 北京市朝阳区人民法院(2018) 京 0105 民初 9840 号民事判决书; 北京互联网法院(2019) 京 0491 民初 6694 号民事判决书。

〔17〕 《民法典》对侵害与损害的区分及其意义,参见程啸《中国民法典侵权责任编的创新与发展》,《中国法律评论》2020 年第 3 期,第 48 - 49 页。

(C)1994-2022 China Academic Journal Electronic Publishing House. All rights reserved.　http://www.cnki.net

显然没有达到《民法典》第1138条第1款要求的"严重精神损害"的程度。倘若要求侵害个人信息权益造成被侵权人严重精神损害时，被侵权人才有权要求侵权人承担精神损害赔偿责任，显然不利于保护个人信息权益遭受损害的被侵权人。故此，《个人信息保护法》第69条第2款在《民法典》第1182条的基础上作了特别规定，将"按照被侵权人因此受到的损失或者侵权人因此获得的利益赔偿"的规则适用于包括财产损失与精神损害在内的所有损害。

七、结语

《民法典》与《个人信息保护法》以保护个人信息权益，实现个人信息保护与利用的协调为目的，二者都是个人信息保护法律体系中重要的组成部分。由于《民法典》是民事领域的基础性法律，其调整的是平等主体的自然人、法人和非法人组织之间的人身关系和财产关系。故此，《民法典》对个人信息权益的规定侧重于权益的内容和侵害等。《个人信息保护法》作为个人信息保护领域的专门性法律，调整的是信息能力不对等的个人信息处理者与个人之间的个人信息处理活动，而且采取了民法、行政法、刑法、国际法等多个部门法的方法进行全方位的调整，故此，《个人信息保护法》不仅侧重于对侵害个人信息权益的事后救济，而且注重对个人信息权益的事先的、预防性的保护。这种调整范围和调整方法的差别使得《民法典》与《个人信息保护法》的关系变得复杂，不能简单地以一般法与特别法的关系或者并存的基本法的关系加以概括，而应当从前文所述的四种类型的角度逐一考察。唯其如此，才能构建科学合理、符合我国国情的个人信息保护法律体系，并在司法和执法中准确适用《民法典》与《个人信息保护法》处理非法的个人信息处理活动与个人信息权益纠纷，最终实现保护个人信息权益，促进个人信息合理利用的目标。

（本文责任编辑　焦和平　肖新喜）

(C)1994-2022 China Academic Journal Electronic Publishing House. All rights reserved.　http://www.cnki.net
