# Supplementary material for: FAIR human neuroscientific data sharing to advance AI driven research and applications: Legal frameworks and missing metadata standards
Source: Front Genet. 2023 Mar 13;14:1086802. doi: 10.3389/fgene.2023.1086802 (PMC10065194; doi:10.3389/fgene.2023.1086802)
Supplement: Supplementary file 2 [file Presentation2.pdf]

·民法典专题·

敏感个人信息保护的基本问题

——以《民法典》和《个人信息保护法》的解释为背景

王利明*

内容提要：《个人信息保护法》采取了“概括+列举”的方式，首次对敏感个人信息的概念作出了规定，并明确规定了“敏感性”的核心特征，将未成年人的个人信息纳入敏感个人信息的范畴，从而区分了敏感个人信息与一般个人信息。敏感个人信息与私密信息隐私存在交叉重合关系，但两者存在一定的区别，在保护方式上应当区别对待。对敏感个人信息的判断主要应依据法定标准，但也有必要兼采“场景理论”。敏感个人信息的处理应遵循“特定目的+单独同意”规则。只有在符合这些条件的前提下，才能对敏感个人信息进行处理。

关键词：个人信息；敏感个人信息；个人信息保护法

敏感个人信息保护是随着互联网、高科技的发展而出现的新问题。在欧盟《一般数据保护条例》（以下称GDPR）中，敏感个人信息被称为“特殊类型的个人数据”（Special Categories of Personal Data）。美国联邦《消费者数据隐私与安全法令》以及有关州将其称为“敏感个人信息”或“敏感数据”。日本《个人信息保护法》第2条将敏感的个人信息称为“需注意的个人信息”。由于敏感个人信息直接关系到个人人格尊严和人身、财产安全等重大权益，因此，需要将个人信息区分为一般个人信息与敏感个人信息，并对敏感个人信息设置特殊的保护规则。我国《民法典》虽然规定了私密信息隐私的保护规则，但并没有对敏感个人信息作出明确规定。我国《个人信息保护法》在借鉴比较法经验的基础上，立足中国实践，设立专章规定了敏感个人信息的处理规则，从而构建了完备的个人信息保护规则，并弥补了《民法典》对个人信息保护的不足。由于敏感个人信息保护是一项新型的制度，涉及一些重大问题需要探讨，本文拟从敏感个人信息的概念出发，就敏感个人信息涉及的相关问题作出初步的探讨。

一、敏感个人信息的概念界定

所谓敏感个人信息（Sensitive Personal Information），是指一旦该信息被泄露或者被非法使用，就容易导致自然人的人格尊严受到侵害或者人身、财产安全受到危害的个人信息。早在1970年，德国黑森邦的《个人信息保护法》中，就出现了敏感数据的概念，有德国学者将其界定为具有极高个人属性、对识别个人身份十分重要的、有受损害或者歧视风险的信息，其标识着个人某项特定的属性。[1]《1981年欧洲委员会个人数据公约》第6条规定，除非国内法提供适当的保障，否则不得自动处理有关种族、政治观点、宗教或其他信仰、健康或性生活的个人资

* 中国人民大学民商事法律科学研究中心研究员、法学院教授，博士生导师，法学博士。

〔1〕Vgl. Paal/Pauly/Frenzel DS-GVO Art. 9 Rn. 6-8.

(C)1994-2022 China Academic Journal Electronic Publishing House. All rights reserved.　http://www.cnki.net

料。上述规定亦适用于与刑事定罪有关的个人数据。[2] GDPR 第 9 条详细列举了敏感个人信息的类型，其包括种族或民族背景、政治观念、宗教或哲学信仰、工会成员身份、基因数据、生物性识别数据，以及和自然人健康、个人性生活或性取向相关的数据。[3] 巴西《通用数据保护法》第 5 条将个人敏感数据规定为："关于种族或族裔、宗教信仰、政治观点、工会或宗教、哲学或政治组织成员身份的个人数据，与自然人有关的健康或性生活数据、基因或生物数据"。美国在联邦立法层面并未对敏感个人信息进行统一定义，而是在相关的、分别的单行立法中，对部分敏感个人信息类别作出了特别规定。如美国卫生与公众服务部（HHS）制定的《个人可识别健康信息的隐私标准》（Standards for Privacy of Individually Identifiable Health Information），就对健康信息隐私作出了特别保护的规定。可见，各国普遍对敏感个人信息设置了特殊的保护规则。

我国《民法典》虽然没有直接采用敏感个人信息的概念，但对"私密信息"作出了规定（《民法典》第 1034 条第 3 款），其也包含一些敏感个人信息。《民法典》第 1034 条第 2 款在《网络安全法》个人信息概念规定的基础上，增加了生物识别信息、健康信息、行踪信息，这实际上也增强了对敏感个人信息的保护。尤其是《民法典》第 1035 条第 1 款规定个人信息的处理应当征得该自然人或者其监护人的同意，就是对未成年人信息的特别保护。可见，《民法典》已经区分了个人信息的不同类型，为《个人信息保护法》规定敏感个人信息保护规则提供了民事基本法依据。《征信业管理条例》第 14 条规定："禁止征信机构采集个人的宗教信仰、基因、指纹、血型、疾病和病史信息以及法律、行政法规规定禁止采集的其他个人信息。"该条就是对敏感个人信息的特殊保护规则。[4] 最高人民法院于 2021 年 6 月 8 日颁布了《关于审理使用人脸识别技术处理个人信息相关民事案件适用法律若干问题的规定》（以下称《人脸识别司法解释》），也为人脸识别信息的特殊保护提供了审判实践的经验。正是在总结我国立法和司法实践经验的基础上，《个人信息保护法》首次采取了"敏感个人信息"的概念，对敏感个人信息作了更加具体、全面的规定。

《个人信息保护法》为个人信息权益的确认和保障提供了一套专门的规则体系，该法是一个领域立法（field of law），其既包括公法规则，也包括私法规则，具有一定的综合性。但就个人信息保护包括敏感个人信息保护规则而言，大部分规则属于私法规则，《个人信息保护法》中的敏感个人信息保护规则属于《民法典》制度规则的具体化，因而《个人信息保护法》和《民法典》是特别法与一般法的关系。在法律适用层面，如果《个人信息保护法》有特别规定，则应当适用该规定；《个人信息保护法》没有作出特别规定的，则仍然应当适用《民法典》。《个人信息保护法》对《民法典》关于敏感个人信息保护规则的具体化主要体现在以下方面：

第一，《个人信息保护法》第 28 条对敏感个人信息的界定采"概括+列举"的方式。一方面，该条明确界定了敏感个人信息的概念和判断标准，为实践中出现的各种新型敏感个人信息的认定提供了明确判断标准，即应当根据是否容易导致自然人的人格尊严受到侵害和是否危害个人人身、财产安全两个标准予以判断。该标准为在实践中准确认定敏感个人信息提供了基本依据；同时，概括型定义的方式具有足够的弹性和开放性，能够应对互联网时代更迭迅速、层出不穷的信息类型，从而适应社会发展需要。该概念虽然是从敏感个人信息受到侵害时的认定标准出发对敏感个人信息作出的界定，但其也明确了敏感个人信息是与自然人的人格尊严或者人身、财产安全具有密切联系的个人信息。另一方面，敏感个人信息的内涵虽然是确定的（即与自然人的人

[2] Rebecca Wong, *Data Protection Online: Alternative Approaches to Sensitive Data?*, 2 Journal of International Commercial Law and Technology 9, 10 (2007).

[3] 参见 GDPR 第 9 条。

[4] 参见程啸：《个人信息保护的理解与适用》，中国法制出版社 2021 年版，第 258 页。

(C)1994-2022 China Academic Journal Electronic Publishing House. All rights reserved. http://www.cnki.net

格尊严或者人身、财产安全具有密切联系的个人信息），但其外延具有不确定性，如特定身份、行踪轨迹等个人信息在什么情形下构成敏感个人信息，需要结合特定场景来判断，不能一概将其认定为敏感个人信息，而且也有不少信息介乎敏感个人信息和一般个人信息之间。考虑到抽象的认定标准有可能在实践中难以具体操作，立法者需要通过具体列举的方式，为敏感个人信息的判断尤其是在特定的场景下认定敏感个人信息提供基本的判断依据。因此，《个人信息保护法》第28条具体列举了敏感个人信息的类别，包括生物识别、宗教信仰、特定身份、医疗健康、金融账户、行踪轨迹等信息，以及不满十四周岁未成年人的个人信息。此种列举型定义方式保证了敏感个人信息界定范围的可操作性。

第二，明确规定了“敏感性”的核心特征，并界分了敏感个人信息与一般个人信息。依据《个人信息保护法》第28条第2款，敏感个人信息的核心要素在于其敏感性，即“一旦泄露或者非法使用，容易导致自然人的人格尊严受到侵害或者人身、财产安全受到危害的个人信息”。从比较法上看，大多采取对敏感个人信息进行具体列举的方式，而没有规定敏感个人信息的界定标准，也没有对敏感个人信息的特征作出规定〔5〕，只是通过具体列举的方式区分个人信息的不同种类，并提供不同层次的保护措施。〔6〕《个人信息保护法》对敏感个人信息认定的核心要素（即“敏感性”）作出规定，体现了鲜明的中国特色。就敏感个人信息而言，其不仅仅属于已识别和可识别的与自然人有关的个人信息，而且也是涉及个人人格尊严和人身、财产安全的个人信息，区分一般个人信息和敏感个人信息很大程度上是考虑到敏感个人信息涉及个人尊严与生命财产安全，一旦被泄露或非法使用，就会给个人造成难以挽回的损害。〔7〕认定为敏感个人信息后，就可为信息处理者设置特殊的处理义务，也为监管机构保护敏感个人信息提供了法律依据。

第三，强化了对未成年人个人信息的保护。从比较法上来看，《一般数据保护条例》虽然规定了对未成年人个人信息的保护，但没有将未成年人信息纳入敏感信息。〔8〕美国有关未成年人个人信息的保护规定则集中于国会颁布的《儿童在线隐私保护法》（COPPA），尚未采取特别严厉的个人信息保护规则。〔9〕许多国家有关个人信息保护的法律都规定了处理未成年人的个人信息要取得其监护人的同意，只是对未成年人的年龄标准规定并不一致。但从域外法的经验来看，一般没有采纳将未成年人的个人信息一概视为敏感个人信息的制度模式。《个人信息保护法》将不满14周岁的未成年人的个人信息均纳入敏感个人信息的范畴，强化了对未成年人个人信息的保护。一方面，按照《个人信息保护法》第31条规定，处理未成年人个人信息需要取得其监护人同意。个人信息的处理者在处理未成年人个人信息时，一是需要验证个人信息被处理者是否为未成年人，二是需要验证作出同意的是否为该未成年人的监护人。这就是理论上所说的“双重验证义务（vertification）”。〔10〕当然，该条并没有确立双重同意规则（即同时需要未成年人和监护人同意的模式）。另一方面，依据该规则，需要为未成年人的个人信息处理制定专门的个人信息处理规则，这既有利于对未成年人的个人信息与其他主体的个人信息进行必要的区分，防止不同个人信息的混淆，也有利于使个人信息处理者明确其处理未成年人个人信息的义务和责任〔11〕。此外，依据《个人信息保护法》第62条，国家网信部门还应当为未成年人个人信息等敏感个人信息的处理制定专门的个人信息保护规则和标准，这不仅为监管者强化未成年人个人信息保护设

〔5〕参见GDPR第6条、巴西《通用数据保护法》第5条、日本《个人信息保护法》第2条、韩国《个人信息保护法》第23条。

〔6〕Vgl. Spindler/Schuster/Spindler/Dalby DS-GVO Art. 9 Rn. 4.

〔7〕杨合庆：《论个人信息保护法十大亮点》，载《法治日报》2021年8月22日，第6版。

〔8〕参见GDPR第9条。

〔9〕参见15 U. S. C. §§ 6501 et seq.

〔10〕同前注〔4〕，程啸书，第278页。

〔11〕同前注〔4〕，程啸书，第281页。

(C)1994-2022 China Academic Journal Electronic Publishing House. All rights reserved. http://www.cnki.net

置了义务，同时也进一步强化了信息处理者保护未成年人个人信息的义务。

综上所述，敏感个人信息的敏感性特征是区分敏感个人信息与一般个人信息的标准，同时也揭示了法律强化对敏感个人信息保护的价值基础，即为了强化对个人人格尊严和人身、财产安全的保护。明确敏感个人信息的概念和类型，这既是确定敏感个人信息特殊保护规则的前提，也是准确适用敏感个人信息保护规则的重要基础。另外，明确敏感个人信息的概念，也有助于专门为敏感个人信息制定特殊的个人信息处理标准，降低企业个人信息处理的合规成本，从而实现个人信息保护与个人信息合理利用之间的平衡。〔12〕

二、敏感个人信息与私密信息隐私的关系

《个人信息保护法》对敏感个人信息保护规则作出了规定，《民法典》人格权编规定了私密信息隐私的保护规则，有必要对二者进行区分。关于敏感个人信息与私密信息隐私之间的关系，学界一直存在着如下观点：一是交叉重合说。此种观点认为，有些信息是敏感个人信息而非私密信息，有一些信息可以同时构成敏感个人信息与隐私中的私密信息。〔13〕 如英国学者 Waldman 认为，隐私具有私密性、敏感性。〔14〕 二是独立区分说，此种观点认为，二者之间是相互分离的，不存在交叉重合的可能。三是私密信息覆盖说，此种观点认为，敏感个人信息能被私密信息完全覆盖。〔15〕 笔者赞同交叉重合说，因为诸如家庭住址、银行账户、行踪轨迹、医疗健康等信息都同时满足敏感个人信息与私密信息的要件，这些信息是重合的，未经同意而泄露这些信息，既侵害了他人的敏感个人信息，也侵害了他人的隐私权。严格地说，敏感个人信息与一般个人信息（非敏感个人信息）是《个人信息保护法》从规范个人信息处理行为的角度出发，对个人信息进行的一种重要分类，何种信息为敏感个人信息，原则上由法律法规规章或国家标准加以确定。而私密信息与非私密信息的区分则是为了正确区分隐私权与个人信息权益的保护方法，是《民法典》从民事权益保护的角度出发对个人信息进行的分类。然而，何为私密信息，何为非私密信息，其判断标准并不是由法律法规规章或标准加以确定，应当从社会公众所具有的一般认知和价值判断的角度出发，依据个案的具体情形进行判断。有学者认为，在认定私密信息时，主要应当考虑如下因素：社会公众对该信息是否作为私密信息对待的认知；该信息涉及自然人的人身财产权益、人格尊严和人格自由的重要程度；该信息对于维护社会正常交往、信息自由的重要程度如何等。〔16〕 显然，从这些考量的因素可见，敏感个人信息与私密信息隐私是一种交叉重合的关系。因此在侵害一些敏感个人信息的情形下，可能出现《个人信息保护法》和《民法典》的相关规则同时适用的问题。

虽然敏感个人信息与私密信息隐私之间有交叉重合，但是两者的保护规则仍然有以下的区别：

第一，二者的范围并非完全等同。例如，对于部分敏感个人信息，信息主体可能出于特定的目的而愿意公开，或已经进行了公开，此类个人信息虽然仍属于敏感个人信息，但不再属于私密信息。例如，个人的行踪信息属于敏感个人信息，但如果某个人已经以自拍视频的方式将自己的行踪上传到视频网站，即在网上公开了该信息，则其就不再属于私密信息。因此，敏感个人信息并不当然是私密信息。同时，一些非敏感的个人信息，如个人的电话号码、通讯地址、车牌信息

---

〔12〕参见胡文涛：《我国个人敏感信息界定之构想》，载《中国法学》2018 年第 5 期，第 242 页。

〔13〕参见程啸：《个人信息保护中的敏感信息与私密信息》，载《人民法院报》2020 年 11 月 19 日，第 5 版。

〔14〕Ari Ezra Waldman, *Privacy as Trust: Information Privacy for an Information Age*, Cambridge University Press, 2018, p. 19-20.

〔15〕参见房绍坤、曹相见：《论个人信息人格利益的隐私本质》，载《法制与社会发展》2019 年第 4 期，第 108 页；张里安、韩旭至：《大数据时代下个人信息权的私法属性》，载《法学论坛》2016 年第 3 期，第 119 页。

〔16〕程啸：《论我国个人信息保护法中的个人信息处理规则》，载《清华法学》2021 年第 3 期，第 55 页。

(C)1994-2022 China Academic Journal Electronic Publishing House. All rights reserved.   http://www.cnki.net

等，如果个人不愿意公开，即便个人将其在一定范围内（如某个人数很少的微信群）公开，而没有向全社会公开，其仍然属于私密信息，[17] 但此类个人信息并不属于敏感个人信息。可见，私密信息也并不当然属于敏感个人信息。

第二，二者保护的侧重点不同。对于私密信息隐私的保护旨在通过禁止非法披露，以防止私密信息的对外公开。私密信息隐私强调的是私密性，依法不得非法刺探、泄露、公开以及未经明确同意擅自处理等。而敏感个人信息的保护则侧重于强调信息处理要求的特殊性。从《个人信息保护法》的规定来看，对敏感个人信息的保护，主要是通过规制信息处理者对敏感个人信息的处理义务，从而严格限制敏感个人信息的收集，防止信息处理者非法处理他人的敏感个人信息，造成对他人的侵害。《个人信息保护法》将敏感个人信息保护置于第二章“个人信息处理规则”之下，主要是从个人信息处理的角度对私密信息提供保护。在具体的规则上，规定了对敏感个人信息的处理需要取得单独的同意。以及在法律有规定的情况下，同意必须以要式的方式作出，即取得书面同意。[18] 因此，敏感个人信息处理规则侧重于对该信息的合法处理，而私密信息隐私的法律规制侧重于对非法披露他人隐私的消极防御，即要求其他民事主体不得采取某种行为来侵害隐私权，而敏感个人信息规则是一种积极义务的设定，要求特定的个人信息处理者应当积极履行相应的法定义务。

第三，是否具有集合性不同。私密信息隐私一般不会形成集合性的信息（即通过与其他信息结合识别特定自然人的各种信息），其本身是单个主体享有的权益。基于人权保护的原因，许多国家将隐私作为基本人权对待，故一般不允许将隐私作集合化处理。而敏感个人信息在被收集之后，则可能形成集合信息，无论是否经过匿名化处理，其都可能集合成为数据。从比较法上看，许多国家和地区的规范性文件均采用的是个人数据权的表述（right to protection of personal data），且这一术语的使用已基本在欧盟层面的立法中达成了一致。经过匿名化处理之后，其可能形成纯粹的数据，从而实现数据的流通与共享。当然随着现代技术尤其是数字化技术的发展，通过数据的自动化技术处理手段，能够实现各种网络画像、精准画像的生成，并可以广泛传播，这些都是对个人信息自动化处理发展与泛滥的体现[19]。这种个人信息的自动化处理又可能会引发算法歧视、算法黑箱、网络画像的滥用等问题。

第四，从义务主体层面来看，私密信息隐私的义务主体是权利人之外的所有人，法律设置隐私权保护规则的主要目的即在于防止权利人之外的其他主体采用刺探、泄露等方式侵害他人的私密信息。而敏感个人信息的义务主体则主要是敏感个人信息的处理者。法律之所以将敏感个人信息从一般个人信息中区隔出来，就是为了对敏感个人信息的处理者设置特殊的义务，从而强化对敏感个人信息的保护。例如，收集敏感个人信息需要取得个人的单独同意，这有利于克服传统的个人信息收集中同意规则被虚化的问题[20]。

第五，是否涉及监管不同。私密信息隐私的保护主要是由权利人自身寻求法律保护，一般不需要公权力的积极介入。而敏感个人信息保护则有赖于监管部门的积极监管。《个人信息保护法》明确规定敏感个人信息保护规则，并将其作为个人信息保护中重点的、专门的监管对象，目的也在于实现对敏感个人信息保护的有效监管。对敏感个人信息，网信部门还应当为敏感个人信息处理、人脸识别、人工智能等新技术、新应用制定专门的个人信息保护规则和标准。

鉴于敏感个人信息与私密信息隐私存在上述区别，在保护方式上应当对二者进行必要的区

[17]同前注〔15〕，张里安、韩旭至文，第120页。

[18]Paal/Pauly/Frenzel DS-GVO Art. 9 Rn. 21-22.

[19]参见丁晓东：《论算法的法律规制》，载《中国社会科学》2020年第12期，第147页。

[20]参见金耀：《消费者个人信息保护规则之检讨与重塑———以隐私控制理论为基础》，载《浙江社会科学》2017年第11期，第67页。

(C)1994-2022 China Academic Journal Electronic Publishing House. All rights reserved.   http://www.cnki.net

分。笔者认为，首先应当将敏感个人信息区分为敏感的私密信息和敏感的非私密信息。例如，如果将人脸识别信息确定为敏感的非私密信息，可仅适用敏感个人信息规则。敏感的私密信息，因为其既属于隐私权的保护对象，也属于敏感的个人信息保护的对象，既要适用《民法典》第1032条、第1033条隐私权保护规则，也要适用《个人信息保护法》中的敏感个人信息规则。例如，健康信息、基因信息等属于此类情况。虽然《民法典》第1034条第3款确认了私密信息优先适用隐私权而不是个人信息保护的规定，但由于敏感个人信息保护与私密信息隐私存在交叉重合的关系，在二者发生交叉的情形时，就涉及对敏感个人信息的侵害究竟应当优先适用隐私权的规则，还是优先适用《个人信息保护法》关于敏感个人信息的处理规则的问题，对此仍有待于进一步探讨。

三、敏感个人信息的判断标准

敏感个人信息的界定不仅涉及对信息处理者的义务，而且涉及监管者如何制定相应的规则，履行监管职责等一系列问题，还关涉权利人在其敏感个人信息受到侵害时的权利救济问题。敏感个人信息并非使人高度敏感，而是因为其极易导致对信息主体的权益的侵害，因此法律需要对其处理设置特别的规则、提供特殊的保护。但在法律构建个人敏感信息的特殊规则时，首先需要界定何为敏感个人信息，并将其与一般个人信息相区分。大多数国家的数据保护法律，都对敏感数据进行了具体列举。《个人信息保护法》第28条第1款界定了敏感个人信息的概念，确定了判断敏感个人信息的标准。在判断敏感个人信息时，首先应当依据法定标准，同时结合具体的场景进行判断。

（一）依据法定标准判断

此处所说的法定标准，首先是指《个人信息保护法》第28条第1款所确立的标准，其次还需要结合行政法规、司法解释的规定予以确定。例如，《征信业管理条例》第14条将血型等纳入敏感个人信息的范畴；再如，《人脸识别司法解释》将人脸信息作为敏感个人信息保护。这些都是行政法规、司法解释对敏感个人信息的具体规定。依据《个人信息保护法》第28条第1款，敏感个人信息的界定应当采纳如下标准：

第一，人格尊严标准。严格地说，所有的个人信息都涉及个人人格尊严的保护，但敏感个人信息不同于一般个人信息之处在于，其与人格尊严的保护具有密切联系，此类信息一旦被泄露或者非法使用，就极易导致信息主体的人格尊严受到侵害。与人格尊严的紧密联系也是认定敏感个人信息的核心标准。因此，《个人信息保护法》第28条特别指出了敏感个人信息与人格尊严关系密切，将敏感个人信息作为“一旦泄露或者非法使用，容易导致自然人的人格尊严受到侵害或者人身、财产安全受到危害的个人信息”，旨在通过遭受侵害会导致人格尊严损害的标准来对敏感个人信息进行识别。敏感个人信息对自然人的社会人格有着极大的风险，对于敏感个人信息的不当处理，会导致自然人遭受损害，尤其会导致对自然人的歧视和不平等对待。[21] 例如，人体基因的泄露会造成个人在就业、保险等社会活动中遭受各种不公正的歧视。[22] 再如，个人的行踪信息若被公之于众或其人脸等生物识别信息被他人不法收集或处理的，将使信息主体的人身安全受到威胁，人格尊严受到侵害。虽然一般个人信息的保护也彰显了对人格尊严的保护，但一般个人信息不如敏感个人信息（如宗教信仰、生物识别信息等）与人格联系紧密，因而敏感个人信息保护相较于一般个人信息而言，更直接和全面地反映了人格尊严保护的需求。因此，将与人格

〔21〕Vgl. Spindler/Schuster/Spindler/Dalby DS-GVO Art. 9 Rn. 4.

〔22〕参见田野等：《论敏感个人信息的法律保护》，载《河南社会科学》2019第7期，第44页。

(C)1994-2022 China Academic Journal Electronic Publishing House. All rights reserved. http://www.cnki.net

尊严的密切联系作为认定敏感个人信息的标准，将进一步强化敏感个人信息具有的保护人格尊严功能。

在实践中，有些敏感个人信息表面上看似乎与人格尊严没有直接联系，但从本质上看，凡是列入敏感个人信息范畴的个人信息都与个人人格尊严的保护具有直接关联。例如，就财务信息而言，表面上似乎主要涉及的是个人的财产信息，主要着眼于保护个人财产安全。法律保护个人的财务信息，具有防止因他人盗用或者冒用账号密码或者处置权限而直接损及个人的物质性财产权的功能，但对财务信息的特别保护并非仅仅是为了保护个人财产，也是为了维护个人的人格尊严。因为擅自处理个人的财务信息，也会导致信息主体的不安和恐惧，特别是财务信息被非法公开会对个人安全构成威胁，会造成对个人的人格性利益的侵害。因此《个人信息保护法》对于敏感个人信息保护的规则，实际上是对《民法典》人格尊严保护的进一步强化。

第二，人身、财产安全标准。敏感个人信息的泄露或非法使用除了人格尊严可能遭受损害外，还可能导致人身、财产安全遭受威胁。因此，敏感个人信息也应当以人身、财产安全为标准。一是人身安全。如果某些信息与自然人的生命、身体、健康具有密切的联系，且一旦受到侵害，将使个人的人身安全受到损害或者面临重大的风险，则应当将其归入敏感个人信息的范畴。例如，个人的行踪轨迹一旦被披露，就可能使个人受到不法行为人的跟踪，其人身安全也将受到巨大威胁。由于生命、健康是最为重要的法益，“生命重于泰山”，因而，与生命、身体、健康有直接联系的个人信息都应当纳入敏感个人信息的范畴，受到法律的特别保护。二是财产安全。严格地说，法律保护敏感个人信息主要不是为了保护财产，而是为了保护个人的人格尊严和生命、健康的安全，GDPR 中，保护敏感个人信息的目的是以保护个人的基本权利与自由为主，并不直接包括保护单纯的财产安全[23]。这里所说的基本权利除了那些和人格尊严密切相关的基本人格利益（如生命、安全、尊严、不受歧视、不受迫害等）之外，所涉及的财产利益主要是指那些与维护人格尊严和安全密切联系的基本财产利益，如就业、社会保障的权利等[24]。但财产安全也与个人的人身安全有密切的联系，一般而言，对财产安全的重要威胁也会损害信息主体的基本人权。例如，非法泄露他人的银行账户，可能严重威胁个人的财产安全，因而，银行账户信息应当纳入敏感个人信息的范畴。在实践中，因为金融账户等信息的泄露，导致各种电信诈骗活动不断产生，给人民群众造成巨大的财产损失。[25] 因此，其也应当属于敏感性的重要组成部分。严格地说，应当将与财产安全具有联系的个人信息限缩为对个人财产安全具有重要影响的个人信息，而不能将所有与个人财产安全具有关联的信息均视为敏感个人信息。

第三，未成年人标准。《个人信息保护法》将不满 14 周岁的未成年人的个人信息均作为敏感个人信息加以对待。然而，就敏感个人信息的一般规则而言，事实上并非未成年人的所有个人信息的泄露均会导致人格尊严受到侵害或者人身、财产安全受到危害。在比较法上，对未成年人的信息虽然给予特别保护，但一般没有将其纳入敏感个人信息的概念之下，而是另外设置独立的规范。鉴于未成年人的信息一旦泄露，即可能被不法行为人利用来对未成年人实施侵害或对家长进行诈骗。因此，《个人信息保护法》将不满 14 周岁的未成年人的个人信息一概作为敏感个人信息，就强化了对未成年人个人信息的保护，可以说是《个人信息保护法》的一大亮点。同时，依据《个人信息保护法》第 31 条的规定，个人信息处理者处理不满 14 周岁未成年人的个人信息应当取得其父母或者其他监护人的同意，并且应当制定专门的个人信息处理规则。

（二）兼采“场景理论”

---

〔23〕参见 GDPR 第 2 条。

〔24〕参见 GDPR 第 9 条。

〔25〕同前注〔4〕，程啸书，第 259 页。

(C)1994-2022 China Academic Journal Electronic Publishing House. All rights reserved.   http://www.cnki.net

依据“场景理论”对敏感个人信息的界定以及与一般个人信息的界分，应当摆脱“全有或全无”的固定思维模式，应当根据个人信息处理行为发生的具体场景，对围绕该行为的各个元素（如行为人、信息主体的身份，处理的目的，处理的场所及其影响的后果等）进行综合评价，确定某信息处理行为的对象是否属于敏感个人信息。因为信息敏感性不是天然存在的，任何信息基于具体的场景都有可能具有敏感性,〔26〕依据该理论，对信息的处理应尊重信息提供时的场景，信息处理者针对相应信息的后续处理行为必须与该场景相一致。〔27〕该理论最初由美国学者 Nissenbaum 教授提出，并为许多国家和地区的学者所采纳，其基本观点认为应当跳出对个人信息的公开与非公开、敏感与非敏感个人信息的简单二分法的窠臼，是否构成敏感个人信息，需要考虑所处情境才能作出判断。〔28〕据此，判断某一信息处理行为是否属于对敏感个人信息进行处理的行为时，必须将信息处理者的目的、处理的环境等场景因素纳入考量，对整个的情景进行综合并且全面的考察。〔29〕从比较法的角度来看，尤其是欧盟与美国的个人信息保护实践，它们对个人信息的保护接近于场景化的行为主义规制。〔30〕在德国，对于宗教信仰、政治观点等个人信息是否视为敏感数据，通常要根据特殊场景考量，来予以确定〔31〕。

虽然我国对敏感个人信息的判断应当采用法定标准，但这显然是不够的，还应当依据具体的情形，来判断敏感个人信息，并将其与一般个人信息相区分。之所以应当兼采场景理论，原因在于：

第一，敏感个人信息的外延具有一定的不确定性，且与个人信息的敏感度是不同的，敏感个人信息具有高敏感度，其遭受泄露或被非法利用可能导致人格受损、引发对信息主体的歧视和妨害其人格尊严，这是对敏感个人信息进行特殊保护的根本原因。〔32〕敏感个人信息的范畴会随着社会变迁而不断发展，范围具有不确定性，需要结合具体场景判断具体范围，而且在不同的场景中，敏感个人信息的范围也存在区别。例如，将他人的家庭住址、电话号码在小范围内公开，与将这些信息放到网络上进行处理，情形是完全不同的，对判断是否构成侵害个人敏感信息产生不同的影响。因此，在具体判断敏感个人信息时，还需要兼采场景理论。

第二，敏感个人信息的范围不断变化。由于法律的列举总是十分有限的，大量的新型的敏感个人信息在产生之后，还需要结合具体场景进行判断。敏感个人信息是一个动态的概念，其范围在不断地发展变化。《个人信息保护法》第 28 条第 1 款在对敏感个人信息的具体列举中使用了“等”字，这就意味着，敏感个人信息并不局限于生物识别、宗教信仰、特定身份、医疗健康、金融账户、行踪轨迹这几种类型，其范围具有开放性。将来随着社会发展将会出现一些新的敏感个人信息类型，敏感个人信息类型的多样化是科学技术和社会发展的必然结果，这就需要法官根据个案判断新类型的个人信息是否属于敏感个人信息，《个人信息保护法》采用开放式列举的方法规定敏感个人信息的范围，为这种适用范围的扩张创造了空间。〔33〕

第三，在对用户进行“用户”画像的情形下，被收集的信息碎片是否构成敏感个人信息，需要结合具体场景进行判断。所谓画像（profiling），就是抓取个人的碎片化信息，然后运用自动化的信息处理技术，来评估个人的兴趣偏好与消费取向等信息。数据画像已经在实践中得到了广泛运用。但数据画像也涉及对敏感个人信息的处理。根据 GDPR 第 4 条第（4）项，即便是匿名

〔26〕See Rebecca Wong, *Supra* note〔2〕, p. 13.

〔27〕See Helen Nissenbaum, *Respecting Context to Protect Privacy: Why Meaning Matters*, 24 Science and Engineering Ethics 831 (2018).

〔28〕See Helen Nissenbaum, *Privacy as Contextual Integrity*, 79 Washington Law Review 119 (2004).

〔29〕Vgl. Paal/Pauly/Frenzel DS-GVO Art. 9 Rn. 6.

〔30〕参见丁晓东：《个人信息保护：原理与实践》，法律出版社 2021 年版，第 86 页。

〔31〕See Rebecca Wong, *Supra* note〔2〕, p. 13.

〔32〕同前注〔12〕，胡文涛文，第 241 页。

〔33〕Vgl. Paal/Pauly/Frenzel DS-GVO Art. 9 Rn. 7.

(C)1994-2022 China Academic Journal Electronic Publishing House. All rights reserved. http://www.cnki.net

的画像，当其被用来针对指涉的人群而作后续决策时，也应该被归为敏感数据。[34]《个人信息保护法》并不禁止数据画像，但是数据画像的后果仍然受到该法有关个人敏感信息规则的约束和规范。在对用户进行“画像”的情形下，信息处理者会收集个人大量的碎片化的信息，这些碎片化的信息都属于用户“画像”的组成部分，但是否涉及敏感个人信息的处理，应当根据具体场景来判断。例如，单纯了解个人的酒店住宿信息，或者购买机票的信息，可能并不构成敏感个人信息，但如果将这些信息组合在一起，则可能形成用户行为轨迹的完整链条，构成敏感个人信息，从而应当适用该法有关个人敏感信息的保护规则。

如何依据场景具体判断敏感个人信息，有学者认为，判断敏感个人信息，需要考虑泄露该信息是否会导致重大伤害，信息的敏感度指信息内容的泄露是否容易对他人造成伤害或影响，泄露该信息给信息主体带来伤害的几率等。此种观点不无道理，但在判断场景时，仍应当结合法定标准，依据具体的场景考虑某种信息与人格尊严、人身、财产安全存在密切关联，具体场景需要特别考虑造成损害的可能性、现实性以及可能造成损害的程度。某些个人信息虽然在一般情形下并不属于敏感个人信息，但此类个人信息一旦与相关的技术手段相结合，也可能对人格尊严造成风险，因此，在特定场景下，其也可能属于敏感个人信息。[35] 例如，个人通讯录中的好友信息，与人格尊严的关系并不密切；而个人的通信内容与通信记录则与人格尊严的关系较为密切。再如，个人的电话号码本身不能够彰显人格尊严价值，但若电话号码被泄露，很可能会导致个人的私生活安宁受到侵扰，此时就关涉信息主体的人格尊严保护问题。此外，在判断是否构成敏感个人信息时，应当结合社会公众的一般观念，而非当事人的主观感受来具体判断。

四、敏感个人信息处理中的“特定目的+单独同意”规则

《个人信息保护法》规定敏感个人信息，并不意味着要在法律责任方面对敏感个人信息与一般个人信息进行区别，实行特殊保护，而在很大程度上是为敏感个人信息的处理确定特殊规则。依据 GDPR 第 9 条，原则上禁止处理敏感数据，除非数据主体明确同意基于一项或多项具体目的而授权处理其个人数据，GDPR 还列出了在不经数据主体明确同意的情况下可以处理敏感数据的几种情况，但前提是有适当的与特定的保障措施，并尊重数据最小化原则[36]。我国《个人信息保护法》对个人敏感信息的处理设置了特殊的规则，明晰了个人信息处理者的义务，明确其在收集个人的敏感个人信息时所应满足的条件、可以处理的范围。《个人信息保护法》第二章专门就敏感个人信息的处理规则作出规定，主要围绕着“特定目的+单独同意”规则而展开，从而进一步强化对信息主体个人信息的保护。

（一）符合特定目的（specific purposes）规则

根据《个人信息保护法》第 28 条第 2 款的规定，只有在具有特定的目的和充分的必要性，并采取严格保护措施的情形下，个人信息处理者才能处理敏感个人信息。这一条款规定了处理敏感个人信息的前提条件：一是具有特定的目的，二是具有充分的必要性，三是采取严格保护措施。其中，这几个要件的核心是特定目的。只有具有特定目的，才能依法处理敏感个人信息，而充分的必要性和采取严格保护措施都是围绕特定目的而具体展开的。法律之所以要求对敏感个人信息的处理必须符合特定目的，其原因在于，敏感个人信息具有不同于一般个人信息的敏感性，

〔34〕See Rebecca Wong, *Supra* note〔2〕, p. 10.

〔35〕Vgl. Paal/Pauly/Frenzel DS-GVO Art. 9 Rn. 7.

〔36〕Nadina Iacob & Felice Simonelli, *Towards a European Health Data Ecosystem*, 11 European Journal of Risk Regulation 884, 889 (2020).

(C)1994-2022 China Academic Journal Electronic Publishing House. All rights reserved. http://www.cnki.net

直接关系到个人的人格尊严和人身财产安全。与敏感个人信息相比，法律更加注重对一般个人信息的利用及其经济效用的发挥，而对敏感个人信息而言，法律并不鼓励对敏感个人信息的利用。例如，依据 GDPR 第 9 条规定，原则上禁止处理个人的敏感数据，只有在该条规定的特殊情形下，才能处理个人的敏感数据。一般认为，该条第 2 款所规定的第（a）项至第（j）项是有穷列举，在对这些情形进行解释时，必须采取严格解释（are to be interpreted restrictively）规则。[37] 这表明，对“特定的目的”不能泛泛而谈，而必须由法律法规明确加以规定。依据该条规定，基于欧盟或会员国法律或集体协议授权，根据会员国法律，为数据主体的基本权利提供适当的保障，可以处理个人的敏感个人信息。基于此种立法价值取向上的区别，法律对敏感个人信息采取的是“特定目的+单独同意”的规则，这也是敏感个人信息与一般个人信息的本质区别。

然而，“特定的目的”是不确定概念，其语义存在较大的解释空间，有必要进一步界定和进行类型化分析。《个人信息保护法》第 6 条规定了个人信息处理的目的限制原则，即任何个人信息的处理都应当具有明确、合理的目的。而依据《个人信息保护法》第 28 条，对敏感个人信息的处理还应当具有特定的目的，也是合法处理敏感个人信息的前提和基础。这也意味着，只有具有特定的目的，才能进一步取得个人同意，并在此基础上处理敏感个人信息。《个人信息保护法》第 28 条关于敏感个人信息处理的特定目的规则，在广义上属于第 6 条所规定的明确、合理目的的范畴，也就是说，第 6 条关于个人信息处理应当具有明确、合理目的的规定，也适用于敏感个人信息的处理。但二者之间存在一定的区别，特定目的标准要高于明确、合理目的标准。笔者认为，应当从如下几个方面理解敏感个人信息处理中的“特定的目的”：

第一，特定的目的必须是特定化的、具体的、明确的目的，而不是泛泛的目的。例如，医疗研究机构、药品开发机构在收集个人的基因信息时，不能笼统地以符合医疗健康的目的而收集他人的基因信息，而应当明确指明其收集他人基因信息的具体目的，如制造某种药品防止基因突变，或者为了研究某种疫苗而收集他人的基因信息。信息处理者收集他人敏感个人信息的目的越具体，也就越有利于确定该目的是否具有充分的必要性。需要指出的是，所谓的特定化的、具体的、明确的目的，主要是遵照立法机构或执法机构所确定或指定的目的处理敏感个人信息[38]，或者是个人明确同意基于某种特定目的而处理其敏感个人信息。这种“一揽子”授权、概括同意等方式都不符合特定目的原则，容易诱使信息处理者在信息主体没有实质上知悉和同意的情况下处理敏感个人信息，从而有损于自然人的人格尊严，或威胁人身、财产安全。

第二，特定目的可以是立法机关和执法机关明确指定的目的。基于立法机关指定的目的，信息处理者可以依法处理个人的敏感个人信息。例如，在相关的疫苗研发中，有关医疗机构可以基于立法或者执法机关的授权，处理个人的敏感个人信息。从比较法上，有的立法也采取了此种立场。将此处的特定目的解释为可以是立法机关或者执法机关明确指定的目的，也有利于对处理敏感个人信息的行为进行事前监管，可以有效地节省信息成本。一方面，信息处理者可以清楚地知悉其处理敏感个人信息的边界，节省其在合规中的信息成本。另一方面，信息主体同样也可以明确地知道自己的敏感个人信息被处理的边界，一旦信息处理者超越这些指定的目的处理敏感个人信息，信息主体就可以主张删除权等请求权，同样也具有节省信息处理成本的优势。

第三，特定目的的判断应当与充分的必要性相结合。依据《个人信息保护法》第 28 条第 2 款的规定，处理敏感个人信息应当同时具备特定的目的和充分的必要性，二者之间具有不可分割的关系，此处所说的充分必要性应当是为实现特定目的的必要性和不可或缺性。对一般的个人信

〔37〕Christopher Kuner, Lee Bygrave & Christopher Docksey eds., *The EU General Data Protection Regulation (GDPR): A Commentary*, Oxford University Press, 2020, p. 375.

〔38〕同前注〔4〕，程啸书，第 267 页。

(C)1994-2022 China Academic Journal Electronic Publishing House. All rights reserved. http://www.cnki.net

息而言，其处理具有必要性即可，而处理敏感个人信息则需要具有充分的必要性，这也体现了对敏感个人信息的强化保护。例如，APP 会收集用户的性别信息，如果将性别纳入敏感个人信息的范畴，则收集用户的性别信息就不再具有充分的必要性。再如，在银行开卡购买理财产品，银行要收集用户的宗教信仰信息，也不具有充分的必要性。充分必要性的判断也与处理个人信息的目的具有直接关联。例如，如果保姆需要与家庭成员密切接触，则需要收集保姆的健康信息；但如果只是聘请一般的保洁员，与家庭成员并没有接触，则收集其健康信息就不再具有充分的必要性。

第四，特定目的的判断不能泛化，在判断特定的目的时，还要考虑信息处理者的职业、活动性质，以及处理敏感个人信息是否出于应对突发事件，是否是为了实现公共利益等因素。在出现上述情形时，有必要对个人的信息权利进行必要的限制，即应当允许信息处理者处理个人的敏感个人信息。当然，即便在上述情形下，信息处理者也应当在必要的范围内处理个人的敏感个人信息，否则仍然构成非法处理个人信息的行为。有的立法也采取了此种立场，例如，依据 GDPR 第 9 条规定，如果是为了实现公共利益，则可以在必要的范围内处理个人的敏感个人信息。

（二）符合单独同意规则

敏感个人信息的处理与一般个人信息的不同之处在于，在符合特定目的的情形下，还应当取得个人的单独同意授权，不允许通过默示授权的方式作出同意。在日常生活中使用各类 APP 时，信息处理人往往通过一揽子授权的方式获得信息主体的授权。许多 APP 均没有逐项取得消费者单独同意，便开始收集敏感个人信息。〔39〕《人脸识别司法解释》已经针对此种现象在第 2 条第 3 项规定了将未取得单独同意作为侵害人格权益的行为。个人信息的一般处理规则是基于信息主体的同意，而敏感个人信息的处理要求“只有在具有特定的目的和充分的必要性，并采取严格保护措施的情形下，个人信息处理者方可处理敏感个人信息”。并且，对于敏感个人信息的同意和告知事项，《个人信息保护法》都作出了更严格的规定，要求处理敏感个人信息应当符合单独同意规则。作出此种规定的原因在于，一方面，进一步提高了对信息处理者的义务要求，通过单独授权的方式，严格限制敏感个人信息的收集，以防止对敏感个人信息的侵害。另一方面，对信息主体而言，单独授权的方式也可以使其意识到授权所引发的风险，从而谨慎授权，并强化权利保护意识。〔40〕

《个人信息保护法》第 29 条规定：“处理敏感个人信息应当取得个人的单独同意；法律、行政法规规定处理敏感个人信息应当取得书面同意的，从其规定。”该条提高了个人信息保护中的知情同意原则的要求，依据该规定，对于敏感个人信息处理的同意必须是“单独同意”。那么应当如何理解“单独同意”呢？笔者认为，可以从以下三个方面加以理解：

第一，“单独同意规则”禁止一揽子授权。〔41〕对于各种敏感个人信息的处理，需要逐项取得信息主体的授权。但在实践中，信息处理人往往通过概括授权的方式，取得信息主体的同意。而在概括授权的模式下，信息主体往往并不知晓其授权的具体内容，以及是否包括敏感个人信息。依据《个人信息保护法》的规定，敏感个人信息的处理应当采用单独同意规则，信息处理者需要单独向个人告知处理敏感个人信息的必要性以及对个人权益的影响，并取得个人明确的同意。《人脸识别解释》第 4 条第 2 项也规定，信息处理者以与其他授权捆绑等方式要求自然人同意处理其人脸信息的，无法产生取得用户同意的效力。虽然在法律、法规没有明确要求时，并不

〔39〕参见南都个人信息保护课题组：《南都实测：多款 App 收集人脸等敏感信息未获单独同意》，《南方都市报》2021 年 4 月 30 日，第 6 版。

〔40〕同前注〔4〕，程啸书，第 273 页。

〔41〕Vgl. Gola/Schulz, DS-GVO Art. 9 Rn. 16 f.

(C)1994-2022 China Academic Journal Electronic Publishing House. All rights reserved.   http://www.cnki.net

以书面的形式为必要，但也绝不能是概括的，也不能是隐晦或者默示的。[42] 在特殊情形下，依据法律、法规的规定，不仅需要取得个人的单独同意，还应当取得个人的书面同意，这进一步表明对敏感个人信息处理的严格限制。《个人信息保护法》第 29 条同时规定，法律、行政法规规定处理敏感个人信息应当取得书面同意的，从其规定。例如，《征信业管理条例》第 14 条第 2 款规定："征信机构不得采集个人的收入、存款、有价证券、商业保险、不动产的信息和纳税数额信息。但是，征信机构明确告知信息主体提供该信息可能产生的不利后果，并取得其书面同意的除外。"

第二，"单独同意规则" 要求处理者负有明确告知义务。[43] 在单独授权中，敏感个人信息以逐项授权方式进行，信息主体可以单独就某项信息的处理要求信息处理人应当如实告知被处理信息的范围以及用途，如此才能确保信息主体的知情同意。对于单独同意的理解应当与特定目的相结合，依据《个人信息保护法》第 30 条，信息处理者处理敏感个人信息的，除了负有处理一般个人信息的告知义务之外，还应当告知具有特定目的和充分必要性，而不是泛泛告知处理的一般目的。同时，信息处理者要告知处理敏感个人信息对个人权益产生的影响，使信息主体意识到授权的后果，从而理性地作出是否授权的决定。

第三，"单独同意规则" 应当贯彻拒绝提供服务的限制规则。在实践中，一些 APP 运营商虽然会弹出显眼的提示来询问用户是否同意运营商对其个人信息进行处理，但是在遭到用户的拒绝时，该 APP 往往会强制退出，拒绝向用户提供服务。例如，拒绝提供行踪信息，就直接退出 APP 无法使用。这种行为也损害了信息主体的合法利益。对此，《个人信息保护法》第 16 条已经对信息主体不同意授权时处理人拒绝提供服务的问题进行了限制。而单独同意规则将敏感个人信息以逐项授权的方式进行，而不再采用一揽子授权，这就可以有效实现对拒不提供服务的限制。在信息主体提供了处理人提供服务所必要的信息后，虽然对于部分敏感个人信息没有提供授权，但不影响提供服务，信息处理人不能再以缺乏一揽子授权为由拒绝提供服务。《人脸识别规定》第 4 条第 1 项规定：如果信息处理者要求自然人同意处理其人脸信息才提供产品或者服务的，信息处理者以已征得自然人或者其监护人同意为由抗辩的，人民法院不予支持，但是处理人脸信息属于提供产品或者服务所必需的除外。这也进一步明确了信息处理者不得以拒绝提供服务为条件来强制收集他人的敏感个人信息。

结　语

《个人信息保护法》关于敏感个人信息处理规则的规定，是进一步强化个人信息保护的具体举措和重要亮点，也是对《民法典》关于个人信息保护制度的重大发展。强化对敏感个人信息的保护，需要加强企业合规、政府监管等协同治理，更有待于将敏感个人信息保护的规则与《民法典》的规则相互衔接。由于《民法典》和《个人信息保护法》之间的关系是基本法与单行法的关系，因此，在涉及敏感个人信息的保护时，首先要适用《个人信息保护法》的规定，同时应当注重其与《民法典》的结合。《民法典》所确立的关于人格权请求权、禁令制度、精神损害赔偿等都为个人信息保护确立了基本的救济方法，由于这些规则在《个人信息保护法》中并没有规定，因此，《民法典》的前述规定具有对敏感个人信息实行兜底保护的作用。

责任编辑： 王国柱

[42] Vgl. Spindler/Schuster/Spindler/Dalby DS-GVO Art. 9 Rn. 7.

[43] Vgl. Paal/Pauly/Frenzel DS-GVO Art. 9 Rn. 28.

(C)1994-2022 China Academic Journal Electronic Publishing House. All rights reserved. http://www.cnki.net
